# Wearable Electrochemical Glucose Sensors for Fluid Monitoring: Advances and Challenges in Non-Invasive and Minimally Invasive Technologies

**DOI:** 10.3390/bios15050309

**Published:** 2025-05-12

**Authors:** Ming Wang, Junjie Zheng, Ge Zhang, Shiyan Lu, Jinli Zhou

**Affiliations:** 1 School of Chemical and Printing-Dyeing Engineering, Henan University of Engineering, Zhengzhou 450007, China; jerry_wm@163.com; 2 College of Intelligent Textile and Fabric Electronics, Zhongyuan University of Technology, Zhengzhou 450007, China; 2022110445@zut.edu.cn (J.Z.); 6897@zut.edu.cn (G.Z.); 5341@zut.edu.cn (S.L.)

**Keywords:** non-invasive, minimally invasive, electrochemical, glucose, sensor, biofluid

## Abstract

This review highlights the latest developments in wearable electrochemical glucose sensors, focusing on their transition from invasive to non-invasive and minimally invasive designs. We discuss the underlying mechanisms, performance metrics, and practical challenges of these technologies, emphasizing their potential to revolutionize diabetes care. Additionally, we explore the motivation behind this review: to provide a comprehensive analysis of emerging sensing platforms, assess their clinical applicability, and identify key research gaps that need addressing to achieve reliable, long-term glucose monitoring. By evaluating electrochemical sensors based on tears, saliva, sweat, urine, and interstitial fluid, this work aims to guide future innovations toward more accessible, accurate, and user-friendly solutions for diabetic patients, ultimately improving their quality of life and disease management outcomes.

## 1. Introduction

Diabetes is a common chronic metabolic disease, typically associated with hyperglycemia. When diabetes occurs, the pancreas is unable to effectively produce or utilize insulin, leading to abnormally elevated blood glucose levels [1]. Prolonged hyperglycemia can damage various organ systems, including the cardiovascular system, nervous system, kidneys, and vision. Diabetes is primarily classified into Type I and Type II, with distinct pathogenesis, onset age, and treatment approaches. Type I diabetes is caused by an autoimmune attack on pancreatic β-cells, resulting in insufficient insulin secretion. It usually develops during adolescence and is linked to both genetic and environmental factors. Patients often require insulin injections to control blood glucose levels [2]. In contrast, Type II diabetes is caused by insulin resistance or insufficient insulin secretion, and it typically occurs in older populations. Lifestyle factors, such as healthy eating and increased physical activity, are crucial for managing Type II diabetes [3]. Diabetes has become a global health challenge. According to the World Health Organization, diabetes-related causes lead to 2 million deaths globally each year [4]. This alarming statistic highlights the significant threat diabetes poses to global health.

Traditional diabetes detection methods typically involve blood sampling, requiring a needle to pierce the skin, which may cause pain and pose a risk of infection. This is particularly disadvantageous for children, the elderly, immunocompromised patients, or individuals with needle sensitivity [5]. As a result, developing a non-invasive detection method has become increasingly important. Ma et al. highlight progress in electrochemical sensing, smart drug delivery, and artificial pancreas development, addressing challenges toward achieving painless, intelligent diabetes management for improved patient outcomes [6]. Glucose detection using alternative biofluids such as sweat, tears, saliva, urine, or interstitial fluid shows great potential. This approach not only reduces the pain and infection risks for patients but also enhances comfort and convenience, providing new possibilities for real-time monitoring and management of diabetes [7].

In recent years, with the rapid advancement of medical technologies, wearable devices have demonstrated immense potential in disease monitoring and treatment, particularly showing significant advantages in the non-invasive and minimally invasive monitoring of biological and chemical molecules [8]. The rapid progress of wearable technology has propelled the development of telemedicine [9], and its integration with electrochemical glucose sensors provides diabetes patients with a convenient and comfortable solution for real-time monitoring. Wearable glucose sensors based on biofluids not only play a critical role in smart health management and medical care but also enable precise, real-time, and non-invasive analysis of glucose levels in the body, simplifying traditional detection methods [10].

For patients with Type I diabetes, wearable electrochemical glucose sensors hold particular significance. Since they require frequent blood glucose monitoring and insulin adjustments, real-time data can facilitate more effective blood glucose management. These sensors not only monitor glucose levels but can also simultaneously track physiological signals such as temperature, pulse, and blood pressure, as well as biochemical parameters like dopamine and ions. Continuous glucose monitoring (CGM) systems have played a pivotal role in the development of electrochemical sensors. Compared to traditional self-monitoring blood glucose methods, CGM systems offer greater stability, lower detection limits, and faster response times and are easier to operate and miniaturize [11]. Modern CGM technology has further enhanced accuracy, extended sensor lifespan, and simplified data transmission. Some newer systems no longer require fingerstick calibration, enable longer wear times, and provide real-time monitoring and remote data sharing through devices like smartphones [12]. Moreover, the functionality of CGM systems is gradually expanding; they not only offer alert features but can also integrate with insulin pumps to form closed-loop systems, automatically adjusting insulin infusion rates based on glucose levels [13,14,15,16]. These technological advancements provide diabetes patients with more comprehensive blood glucose management solutions, significantly improving their quality of life.

Research has shown that nanomaterials can enhance the performance of sensors by improving sensitivity, detection limits, biocompatibility, and flexibility. These materials enhance glucose catalysis by providing excellent enzyme immobilization matrices and improving the diffusion of target substances and electrolytes. These materials improve glucose sensing performance by serving as highly effective enzyme immobilization platforms, while simultaneously facilitating the diffusion of analytes and electrolytes [17]. Carbon nanomaterials play a significant role in wearable electrochemical glucose sensors, significantly boosting sensitivity and selectivity due to their exceptional electrical conductivity, mechanical strength, and large surface area [18]. Key carbon materials include carbon nanotubes (CNTs) [19,20,21,22,23,24,25,26,27,28], graphene [29,30,31,32,33,34,35,36,37,38,39,40,41], carbon nanofibers (CNFs) [42,43,44,45,46,47], nanodiamonds (NDs) [48,49,50,51,52,53,54,55,56,57], fullerenes (C60) [58,59,60,61], carbon dots (CDs) [62,63,64,65,66,67], and carbon black (CB) [68,69,70,71,72]. These carbon-based materials serve as ideal platforms for wearable glucose sensing, offering both stable electron transfer and tunable catalytic activity. Their performance can be further optimized via doping (e.g., nitrogen or sulfur incorporation to modulate electronic conductivity) and functionalization (e.g., surface modification with hydrophilic groups or enzyme-anchoring sites), which enhance biocompatibility, selectivity, and interfacial charge transfer in non-invasive/minimally invasive fluid monitoring systems. Wearable glucose sensors leveraging carbon-based nanomaterials (e.g., carbon nanotubes, graphene) offer exceptional biocompatibility, tunable surface chemistry, and high catalytic efficiency—critical for non-invasive/minimally invasive fluid monitoring. These materials, pioneered by Iijima et al. [19], provide large surface areas and strong adsorption capacities, while hybrid systems incorporating metal nanomaterials (e.g., Au, Pt) further enhance electrochemical signal amplification in sweat or interstitial fluid detection. Transition metals and their oxides [73,74,75,76,77], sulfides [78,79,80,81], and phosphides [82,83] are cost-effective and offer excellent electrocatalytic activity and biocompatibility, making them widely used in non-enzymatic sensors. Precious metals such as gold, silver, platinum, and palladium [84,85,86,87,88,89,90,91,92,93,94,95,96,97,98,99,100,101,102,103] enhance sensor sensitivity and selectivity due to their outstanding conductivity and catalytic activity. Additionally, conductive polymer nanomaterials exhibit excellent flexibility, making them suitable for various shapes and structures, with applications in flexible electronics, biosensors, and healthcare [104]. Conductive polymers such as polycarbazole [105,106,107], polyaniline [108,109,110,111,112,113,114,115,116,117,118,119,120,121], polythiophene [122,123,124,125,126,127,128,129,130], and their derivatives have become ideal candidates for wearable electrochemical glucose sensors due to their excellent conductivity, mechanical flexibility, chemical stability, and environmental friendliness. Polycarbazole, with high carrier mobility and stability, aids in glucose detection and chiral recognition. Polyaniline stands out for its low cost, high conductivity, and excellent electrochemical performance, particularly in enzyme loading and enhancing sensor selectivity. Polythiophene and its derivatives, such as PEDOT, improve electrode charge transfer efficiency through their conductivity, stability, and film-forming properties. While the catalytic properties of conductive polymers may be slightly inferior to those of metal nanomaterials, their environmental sustainability makes them highly promising for future sensor development.

In addition to electrochemical methods, light-based glucose sensors (e.g., optical, fluorescence, and plasmonic sensors) have emerged as promising alternatives. These sensors typically rely on glucose-induced changes in optical properties (e.g., absorbance, fluorescence quenching, or surface plasmon resonance) of functional materials [7,89]. For instance, hyaluronate–gold nanoparticle complexes enable wireless glucose detection via near-infrared spectroscopy [89], while carbon dots functionalized with glucose oxidase exhibit fluorescence modulation [67].

However, light-based sensors face challenges such as (a) interference from ambient light and biological matrices (e.g., blood turbidity), which may reduce accuracy [13]; (b) complex instrumentation (e.g., spectrometers), limiting portability [7]; and (c) limited scalability due to high costs of optical components [8]. In contrast, electrochemical sensors offer (a) high sensitivity and selectivity via direct electron transfer (e.g., using carbon nanotubes or graphene) [25]; (b) miniaturization potential with wearable formats (e.g., continuous glucose monitors) [11]; and (c) cost-effectiveness and compatibility with point-of-care devices [12].

While light-based sensors excel in non-invasive applications (e.g., tear or sweat analysis), electrochemical systems dominate clinical settings due to their robustness and quantitative precision [10]. Recent advances in hybrid systems (e.g., electrochemiluminescence) [40] may bridge these technologies.

The integration of conductive materials with non-invasive and minimally invasive technologies, such as microfluidic systems, wearable fabrics, self-powered devices, microneedles, subcutaneous sensors, and microdialysis, has significantly advanced the real-time monitoring of glucose in biofluids. These technologies, by incorporating innovative electrochemical detection mechanisms, enable precise analysis of glucose concentrations in tears, saliva, sweat, urine, and interstitial fluid, thereby providing painless and continuous monitoring solutions. Furthermore, the application of flexible electronics and smart materials enhances the mechanical flexibility and comfort of the sensors, accommodating dynamic changes in various physiological environments. The power supply for flexible glucose sensors is a critical consideration for real-world deployment. Recent innovations include biofuel cells that leverage glucose oxidation for self-powering [5,16], flexible batteries using conductive polymers [104,129], and wireless NFC-based systems [89]. Energy-harvesting mechanisms (e.g., triboelectric nanogenerators) further reduce dependency on external batteries [8]. Future designs may integrate hybrid power systems to enhance reliability. The combination of self-powered technologies and energy harvesting systems extends the device’s lifespan and reduces the need for frequent energy replenishment. These integrated technologies not only address the limitations of traditional invasive monitoring methods but also promote the widespread application of high-performance, intelligent wearable devices in diabetes management, with the potential to significantly improve patients’ quality of life and the convenience of health monitoring.

**Figure 1:**
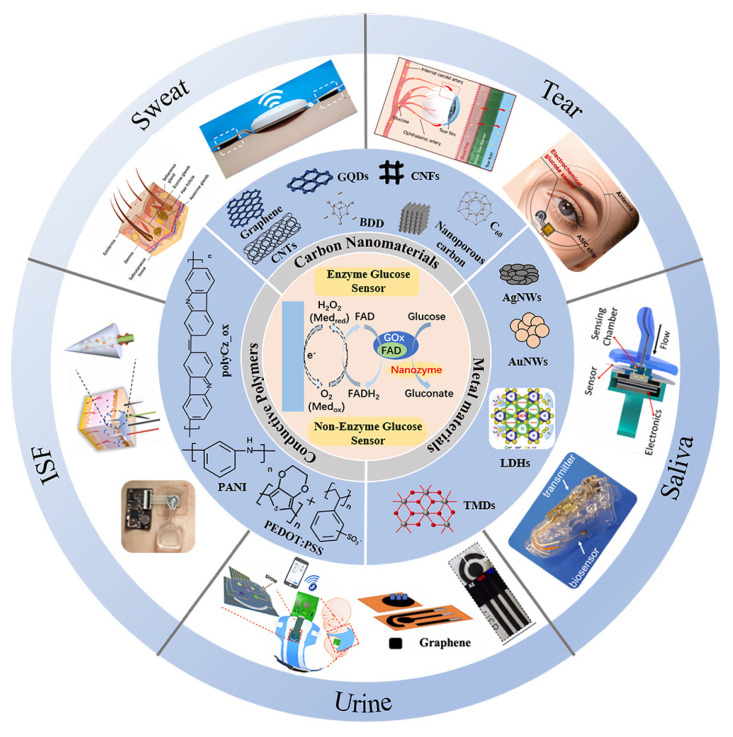
Table of contents (TOC) graphic of this review (clockwise from tear fluid).

This review focuses on the advancements and challenges of wearable electrochemical glucose sensors in non-invasive and minimally invasive technologies. It provides a brief overview of the composition and operating principles of wearable electrochemical sensors and offers an in-depth analysis of non-invasive and minimally invasive techniques for glucose monitoring in biofluids, including microfluidics, wearable fabric sensors, and self-powered technologies for non-invasive methods, as well as microneedles, subcutaneous sensors, and microdialysis for minimally invasive methods. The review summarizes glucose monitoring applications based on different biofluids, discusses the challenges faced by these technologies in practical applications, and explores future directions for the development of wearable electrochemical glucose sensors. See Figure 1.

## 2. Overview of Wearable Electrochemical Glucose Sensors

### 2.1. Electrochemical Sensors

#### 2.1.1. Basic Components of an Electrochemical Sensor

**Figure 2:**
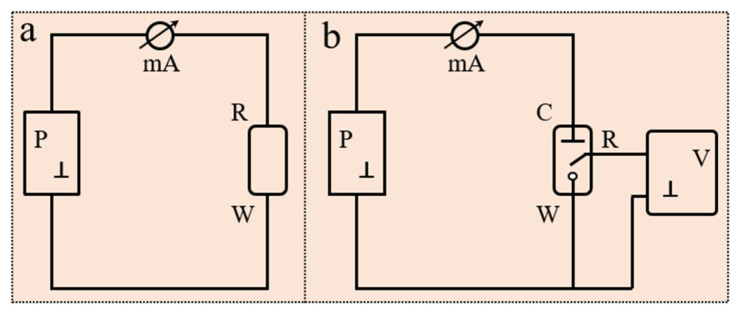
Electrochemical sensor configurations adapted for wearable fabric electrodes: (**a**) simplified two-electrode system; (**b**) conventional three-electrode system with separated RE/CE. Schematics redrawn from fundamental principles [131] with structural optimizations for textile integration (see Section 2.2).

Electrode systems commonly used in electrochemical sensors are typically divided into two types: two-electrode systems and three-electrode systems. The main electrodes include the working electrode (WE), counter electrode (CE), and reference electrode (RE). The WE is the core of the sensor, responsible for detecting electrochemical reactions and generating electrical signals; the CE completes the circuit by facilitating current flow; and the RE provides a stable reference potential. In cases where the current at the WE is relatively small, a two-electrode system, consisting of a WE and RE, can be used. In this configuration, the RE serves both to control the potential and to complete the current loop (Figure 2a, where P is the polarization source). When a larger current flows through the WE, a CE is required to complete the current loop with the WE, while the RE is used solely to control the potential (Figure 2b). In the WE control loop, only a minimal measurement current exists, with no polarization current, while the polarization current mainly flows in the WE-CE loop. Compared to a two-electrode system, the advantage of a three-electrode system lies in its ability to simultaneously control and measure both current and potential, thus minimizing polarization error.

#### 2.1.2. Electrochemical Detection Mechanism

**Electrochemical Detection Mechanisms: Fundamental Principles**

Electrochemical glucose sensors employ four primary transduction mechanisms based on distinct signal types (current, potential, or impedance):

(a) **Amperometry** measures Faradaic current generated by redox reactions at a constant applied potential. The current magnitude follows Fick’s first law of diffusion, where the limiting current (*i*_lim_) is proportional to bulk glucose concentration [131]. The diffusion-controlled electron transfer process is often described by the following equation:

(1) $$i_{lim}=nFAD\frac{C-C_{0}}{\delta }$$

In the equation, i represents the current, n is the number of electrons transferred, F is the Faraday constant, *A* is the effective surface area of the electrode, *D* is the diffusion coefficient, δ denotes the diffusion layer thickness, and *C* and *C*_0_ represent the concentration of the analyte in the bulk solution and at the electrode surface, respectively [131]. Under the condition of diffusion control, the lower the concentration of the analyte on the electrode surface (approaching zero), the greater the diffusion driving force, resulting in the maximum of the limiting diffusion current. At this point, the current response is determined by the diffusion rate and no longer increases with the increase in the potential.

(b) **Potentiometry** quantifies equilibrium potential differences between working and reference electrodes, governed by the Nernst equation for target ion activities [132].

The working principle of potentiometry is to measure the potential difference between the working electrode and the reference electrode, which reflects the concentration of the analyte. Potentiometric sensors do not rely on an externally applied current but instead detect changes in potential caused by the presence of the analyte. According to the standard Nernst equation,

(2) $$EMF=K+\frac{RT}{zF}lna_{1}$$

Here, *EMF* is the electromotive force (potential, observed at zero current), *K* is the constant potential contribution, which includes the liquid-junction potential at the reference electrode, a_1_ is ion activity consisting of charge *z*, and *T*, *R*, and *F* are the absolute temperature, gas constant, and Faraday constant, respectively [132].

Potentiometric sensors measure the potential difference between an indicator electrode and a reference electrode to enable rapid detection of target analyte activity or concentration. They are widely used in environmental monitoring (water quality, soil ion analysis), biomedical applications (blood electrolyte and pH detection), industrial process control (pH monitoring in chemical reactions, food additive testing), laboratory analysis (potentiometric titration, ion-selective detection), and emerging fields (wearable sweat sensors, biomolecule detection). These sensors offer advantages such as high selectivity, real-time response, and miniaturization potential, though limitations include susceptibility to interfering ions and reduced sensitivity at low concentrations (<10^−6^ M) [130].

(c) **Voltammetry** applies potential sweeps to derive concentration–current profiles, with peak currents described by the Randles–Sevcik equation [133].

Voltammetry analyzes analytes in a solution by applying a varying potential to an electrode and measuring the corresponding current response. In biological fluid detection, the following factors require special attention: First, the adsorption of biomolecules on the electrode surface can significantly alter the current response. Second, the high viscosity and complex chemical composition of biological fluids may interfere with the kinetics of the electrochemical reaction. Therefore, systematic optimization and calibration are necessary in practical applications to account for these factors and ensure accurate detection results. According to the Randles–Sevcik equation,

(3) $$I=269000n^{\frac{3}{2}}ACD^{\frac{1}{2}}v^{\frac{1}{2}}$$

In the equation, *n* is the number of electrons transferred in the redox process; *A* is the electrode surface area in cm^2^; *C* is the ion concentration in mol/cm³; *D* is the diffusion coefficient in cm^2^/s; *v* is the scan rate in V/s [133].

(d) **Impedance** spectroscopy analyzes charge transfer resistance and double-layer capacitance through AC frequency responses, modeled by Randles equivalent circuits [5,6]. The principle of impedance spectroscopy is to detect the target analyte by measuring changes in resistance or capacitance at the sensor electrode surface. Impedance Z is typically the ratio of voltage E(t) to current I(t) and is a function of their time-dependent variation. Additionally, the Randles circuit model can be used to describe electrochemical processes in biofluids. The formula is as follows:

(4) $$Z=R_{S}+\frac{1}{j\omega C}+R_{ct}+\frac{1}{Z}$$

In the equation, *R*s is the solution resistance; 1/*jωC* is the double-layer capacitance; *R*ct is the charge transfer resistance; 1/*Z* represents the diffusion impedance.

This model is particularly suitable for simple impedance measurements, especially when the target bioanalyte is present, as the change in charge transfer resistance can be directly assessed by measuring the impedance [5]. Additionally, it is applicable to more complex electrochemical impedance spectroscopy (EIS) analyses [6].

**Challenges from Biofluid Characteristics**

When applied to non-invasive biofluids (sweat/tears/saliva), these mechanisms face inherent limitations, see Table 1:

### 2.2. Electrochemical Glucose Sensor

The modified electrode and transducer are the most critical components of an electrochemical glucose sensor. Sensitive materials are immobilized on the electrode surface through modification, where they specifically bind to the target analyte, triggering a biochemical reaction. This reaction is then converted into an electrical signal by the transducer, which is amplified for measurement, allowing the detection of glucose concentration. Based on whether the modified electrode contains glucose oxidase (GOx), electrochemical glucose sensors can be classified into two categories: enzymatic glucose sensors and non-enzymatic glucose sensors.

#### 2.2.1. Enzymatic Glucose Sensors

**Figure 3:**
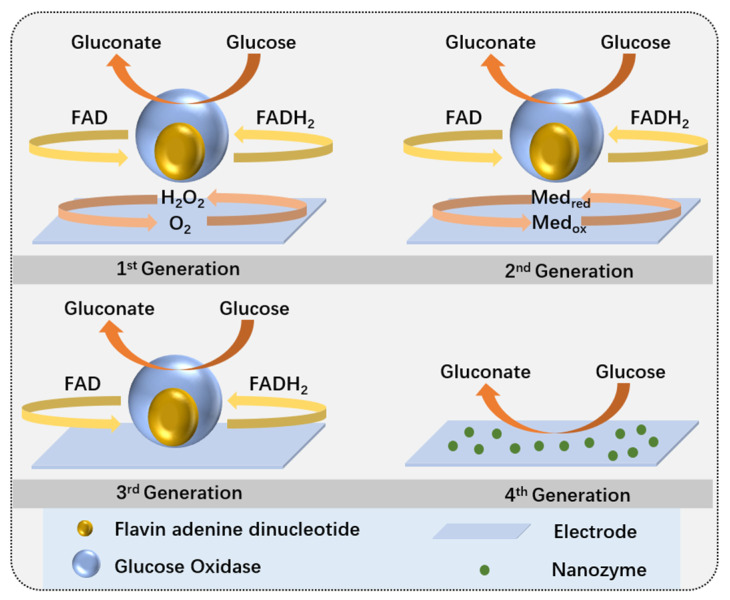
Mechanisms of enzymatic and non-enzymatic glucose sensors [134].

Enzymatic glucose sensors use glucose oxidase (GOx) or glucose dehydrogenase (GDH) as the biological sensing element. These sensors detect glucose levels by measuring the electroactive substances (e.g., H_2_O_2_) produced from the enzymatic reaction with glucose. Currently, enzymatic glucose sensors have evolved to the third generation (Figure 3). The first generation catalyzed glucose oxidation reactions under natural conditions; the second generation used low-molecular-weight compounds to facilitate electron transfer, enhancing measurement range and sensitivity; and the third generation enables direct electron transfer at the redox center and electrode surface without dynamic electron exchange between the enzyme and electrode.

Despite the advantages of high sensitivity and strong selectivity, enzymatic sensors face several challenges in practical applications. For instance, enzymes are susceptible to deactivation due to factors such as temperature, pH, and toxic chemicals. Research has shown that GOx activity is significantly affected or even lost when pH is below 2 or above 8, temperatures exceed 44 °C, or humidity is excessively high or low [134,135]. Moreover, GOx activity can be interfered with by other active substances in biofluids when using mediator molecules [136]. Additionally, most enzyme electrodes are affected by oxygen levels [137]. Due to these limitations, there is increasing interest in non-enzymatic electrodes as alternatives to address these issues.

#### 2.2.2. Non-Enzymatic Glucose Sensors

Non-enzymatic sensors do not rely on enzymes but enhance the catalytic activity of electrodes through direct redox reactions or the use of nanomaterials (such as noble metal nanoparticles and carbon nanotubes) to directly detect glucose. Generally, non-enzymatic glucose sensors are considered to be fourth-generation glucose sensors [134]. Due to their low fabrication costs, long operational lifetimes, simple structures, lack of oxygen dependence, and ease of quality control, non-enzymatic glucose sensors are significant for large-scale production.

### 2.3. Wearable Electrochemical Glucose Sensors

Wearable sensors are integrated analytical devices that combine features of traditional and mobile healthcare, allowing continuous monitoring of personal biological traits in a non-invasive or minimally invasive manner [8,138]. With advancements in wearable technology, the forms of these devices have shifted from traditional formats like watches, shoes, or earphones that track personal health to more compact devices utilizing biological fluids for enhanced comfort and convenience [139,140,141,142,143]. A prominent example is the wearable electrochemical glucose sensor, which uses biological fluids as a medium and relies on biosensing elements and electrochemical transduction components to provide real-time monitoring of glucose levels [144].

**Figure 4:**
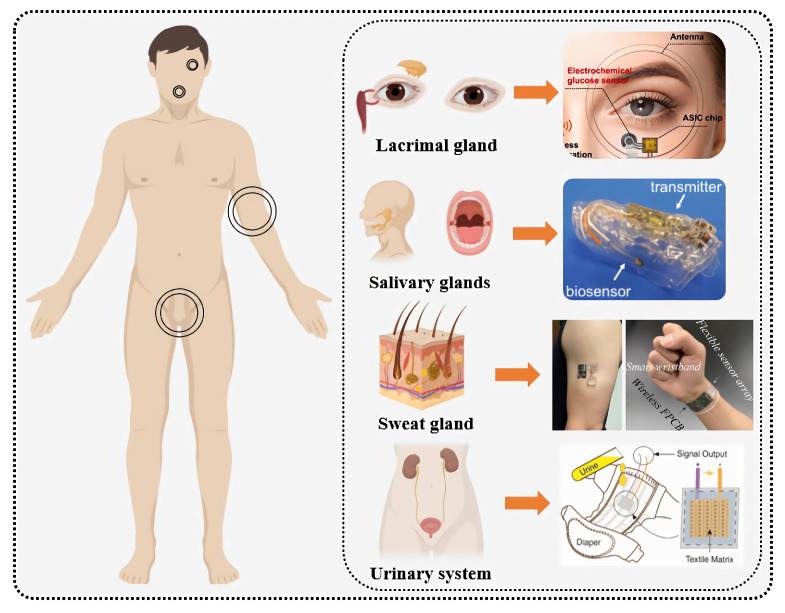
Glands producing biological fluids and related research [141,142,143,144].

Wearable glucose sensors can be categorized based on the type of biological fluid used into non-invasive and minimally invasive types. Non-invasive fluids, such as tears, saliva, sweat, and urine, can be collected without penetration. Minimally invasive fluids, such as interstitial fluid (ISF), which exists in the extracellular space and is closely related to blood circulation, are examples of minimally invasive types. These biological fluids contain trace amounts of glucose, and measuring glucose levels in these fluids can indirectly reflect overall glucose levels. Due to their minimal impact on the body and reduced pain compared to traditional methods, wearable electrochemical glucose sensors based on biological fluids have become a popular topic in the next generation of blood glucose monitoring. See Figure 4.

## 3. Advanced Microfluidic and Wearable Sensing Technologies

### 3.1. Microfluidics

Microfluidics is a technology for manipulating small quantities of fluids within channels on the micrometer or nanometer scale. It achieves fluid mixing, separation, and control through precise regulation of flow rates, pressures, and channel geometries, as well as utilizing interface phenomena and transport effects [145]. This technology is particularly valuable for wearable continuous fluid collection and real-time analysis due to its ability to handle very small fluid volumes (approximately 10^−18^ to 10^−6^ L) in a non-invasive and precise manner.

Common materials used in microfluidics include inorganic materials (glass, silicon, and ceramics), paper, polymers (such as polydimethylsiloxane (PDMS)), and hydrogels [145,146]. The manufacturing of microfluidic devices involves considerations of overall structure, channel layout, fluid control, system integration, and fabrication processes. Optimization of fluid flow and mixing, compatibility with target fluids, and integration with sensors and pumps, as well as ensuring biocompatibility and non-toxicity of the device, are critical aspects. Methods for manufacturing include glass/silicon-based micromachining, polymer-based replication molding, commercial modular assembly, and emerging 3D printing technologies. Niculescu et al. [147] provided a detailed review on material removal and deposition for microfluidic fabrication.

Microfluidic technology holds significant potential for wearable electrochemical glucose sensors by automating the collection and analysis of small fluid samples, enabling non-invasive, continuous, and real-time glucose monitoring [148,149]. This capability is crucial for improving the precision and convenience of health management, reducing user interference, and providing timely health feedback.

However, integrating microfluidic chips into flexible materials presents challenges. The device must maintain flexibility while ensuring biocompatibility and durability to prevent allergic reactions or damage from long-term wear. Additionally, wearable devices often face limitations in fluid collection volumes, and precise fluid control, flow rate management, and sustained flow without external power are significant technical challenges. To meet long-term monitoring needs, systems must operate with low power consumption. Furthermore, the large data volume generated by microfluidic systems requires efficient data processing and transmission to ensure real-time functionality and portability. Lastly, maintaining the stability of microfluidic systems and sensor sensitivity in dynamic environments, influenced by motion, sweat, and temperature changes, is a key challenge in practical applications.

#### 3.1.1. Droplet Microfluidics

Droplet microfluidics, a subclass of microfluidics, involves the generation and manipulation of discrete droplets through immiscible multiphase flows within microchannels. It represents an important branch of microfluidic technology and is widely used in biochemical analysis and material synthesis. Several reviews have discussed the principles of droplet microfluidics [150,151]. The generation of droplets depends on the driving forces and the design of the microchannels and can be classified into two categories: passive methods and active methods [152]. Passive methods generate droplets through natural deformation and break-up of interfaces and include common techniques such as co-flow, cross-flow, and flow-focusing. In co-flow, coaxial channels are used, with droplet size influenced by flow rates and interfacial tension. Cross-flow produces droplets at T-junctions through shear forces, with modes including squeezing, dripping, and jetting. Flow-focusing generates uniform droplets by narrowing the channel, with droplet size affected by channel geometry and fluid properties. These methods do not require external energy input and are suitable for various microfluidic applications. Active methods control droplet formation through external forces and include four main techniques: electric, magnetic, thermal, and mechanical. Electric methods adjust droplet size using electric fields. Magnetic methods generate droplets with magnetic fluids under the influence of magnetic fields. Thermal methods control droplets by altering fluid properties through temperature changes. Mechanical methods produce droplets using hydraulic, pneumatic, or vibrational elements. These active methods offer more flexible control, enabling faster response and precise regulation of droplet formation.

**Figure 5:**
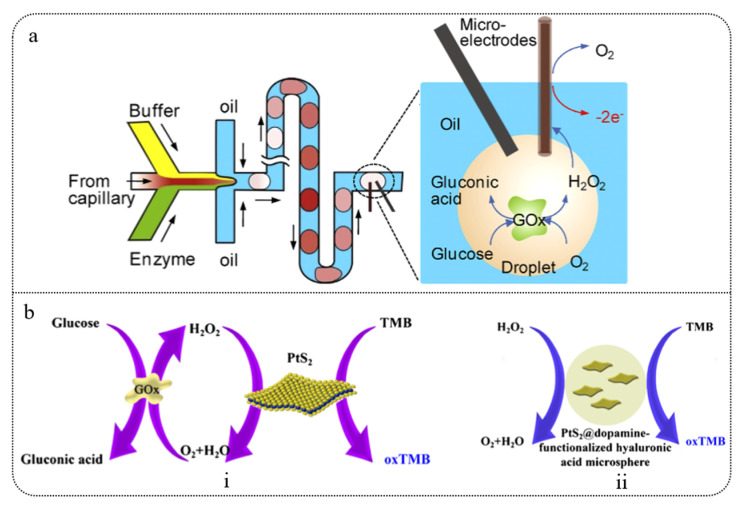
(**a**) Schematic of a microfluidic droplet-based electrochemical sensor and glucose detection principle [153]. (**b**) (**i**) Schematic representation of glucose measurement. (**ii**) Schematic representation of H_2_O_2_ measurement [154].

In the field of glucose monitoring, droplet microfluidics can separate bodily fluids into multiple droplets through tiny channels, integrating with electrochemical sensors to achieve parallel analysis and real-time monitoring of glucose concentrations. Gu et al. [153] developed a droplet microfluidic electrochemical sensor based on platinum black microelectrodes. By electrochemically depositing platinum nanoparticles on bare platinum microelectrodes, they created platinum black microelectrodes that significantly enhanced electrocatalytic activity and current response, showing a 10.2-fold signal enhancement specifically in glucose detection. The glucose detection process is illustrated in Figure 5a. Electrochemical impedance spectroscopy and cyclic voltammetry indicated that platinum black reduced charge transfer resistance and improved electrocatalytic performance towards hydrogen peroxide. The sensor demonstrated high sensitivity and a wide linear range (up to 43.5 mM) in determining GOx enzyme activity and analyzing biological samples, showing potential for application in serum samples. This work provides new insights and technical support for further development of electrochemical sensors in droplet microfluidic systems. Additionally, Zhang et al. [154] developed a peroxidase-like catalytic activity sensor based on PtS2 nanosheets. They successfully prepared PtS2 nanosheets via ultrasonic-assisted liquid exfoliation and demonstrated for the first time that they could catalyze the reaction of H_2_O_2_ with 3,3′,5,5′-tetramethylbenzidine (TMB) to produce a colored product. The schematic of H_2_O_2_ and glucose detection is shown in Figure 5b. The study revealed that PtS2 nanosheets exhibited high stability and catalytic efficiency under a wide range of pH, temperature, and H_2_O_2_ concentration conditions. To enhance the sensitivity and stability of the sensor, the researchers encapsulated PtS2 nanosheets in HA-DA microspheres using droplet microfluidics, designing a H_2_O_2_ sensor based on PtS2 @HA-DA microspheres. By further combining with GOx, they constructed a highly sensitive glucose sensor, successfully applied to glucose detection in human serum, offering new approaches and methods for the use of PtS2 nanosheets in biomedical diagnostics and pharmaceutical analysis.

Theoretically, droplet microfluidics has potential applications in serum samples and other bodily fluids. However, examples of using droplet microfluidics for glucose monitoring in bodily fluids are scarce, with more instances focusing on diabetes treatment and regulation. Araujo et al. [155] developed a dual-drug delivery multifunctional nanocomposite system using droplet microfluidics, which co-loaded glucagon-like peptide-1 (GLP-1) and dipeptidyl peptidase-4 inhibitor (iDPP-4). In vivo experiments conducted in non-obese type 2 diabetes rat models showed that the system could significantly and persistently lower blood glucose levels while prolonging the hypoglycemic effect. Compared to the control group, the hypoglycemic effect increased by 44%, and an increase in insulin levels was observed 6 h after oral administration. Dynamic light scattering and scanning electron microscopy characterized the nanoparticles, revealing that the system effectively reduced blood glucose levels and demonstrated long-term anti-diabetic effects. This study provides a crucial foundation for developing oral protein/peptide delivery systems for Type II diabetes treatment. Li et al. [156] employed droplet microfluidics to develop insulin-rich chitosan (CS) microgels, which were coated with a thin layer of carboxymethyl cellulose (CMC) to form CS@CMC microgels for islet transplantation and long-term glucose regulation. The gel exhibited low immunogenicity, good biocompatibility, and long-term stability in vivo, capable of shielding islet cells from immune proteins and cells while allowing free diffusion of oxygen, nutrients, and insulin. The study successfully achieved glucose regulation in diabetic mice for up to 180 days. The results indicated that CS@CMC microgels could maintain islet cell viability and insulin secretion function without the use of immunosuppressive drugs, showing broad clinical application potential.

#### 3.1.2. Paper-Based Microfluidics

Paper-based microfluidics is another significant branch of microfluidic technology, primarily operating on the principle of using the capillary action of paper or porous membranes to guide fluids. It is highly suitable for portable point-of-care (POC) devices due to its simplicity in fabrication, low cost, ease of use, and non-invasive nature. By integrating colorimetric and electrochemical detection methods, paper-based microfluidics can achieve rapid and quantitative analysis of glucose concentrations in body fluids, demonstrating substantial potential in glucose monitoring. Detailed summaries of the fabrication and measurement methods for paper-based microfluidics can be found in the research conducted by Nishat et al. [157].

**Figure 6:**
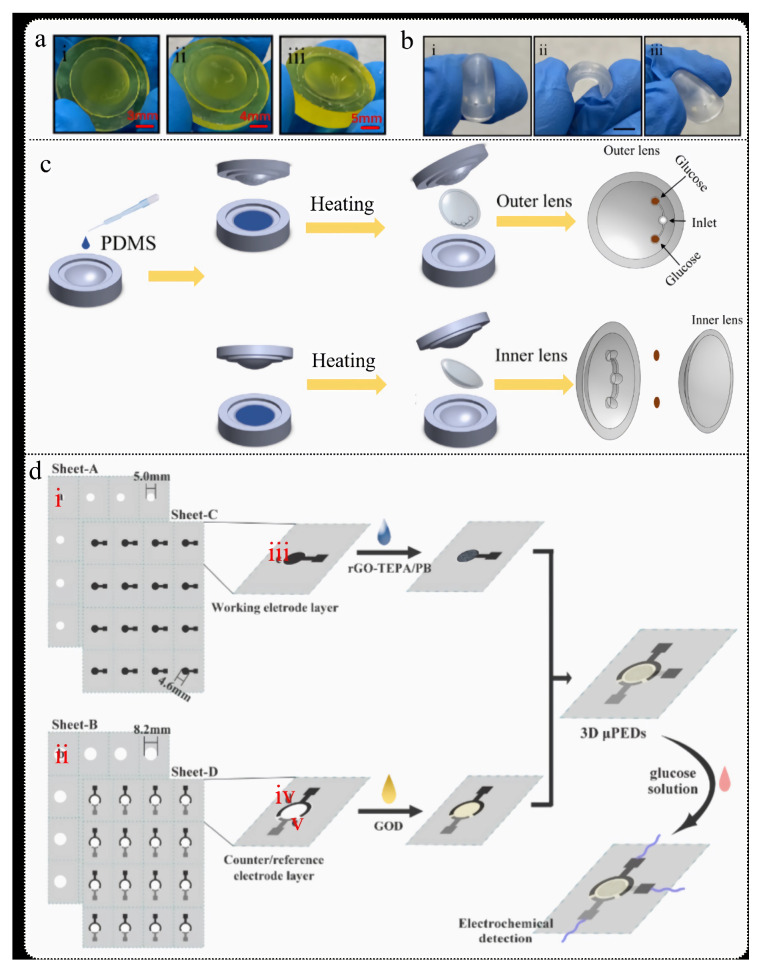
(**a**) Mold containing microfluidic channels: (**i**) schematic, (**ii**) top view, (**iii**) cross-sectional view [158]. (**b**) Schematic diagrams of the soft contact lens (SCL) under mechanical stress, bending, and different angles: (**i**) mechanical stress, (**ii**) bending, (**iii**) different angles [158]. (**c**) Lens molding process [158]. (**d**) A 3D paper-based microfluidic electrochemical biosensor: (**i**) hydrophilic zone, (**ii**) carbon working electrode, (**iii**) carbon counter electrode, (**iv**) carbon counter electrode, (**v**) Ag/AgCl reference electrode [159].

One common approach involves integrating glucose oxidase (GOx) onto paper, where the enzymatic reaction of glucose oxidation generates hydrogen peroxide (H_2_O_2_), and glucose concentrations are reflected by color changes in dyes or reagents. Isgor et al. [158] developed a wearable contact lens made using 3D printing and soft lithography techniques for non-invasive photometric glucose detection (Figure 6a). The contact lens structure consists of three layers: an internal lens for structural support, a colorimetric detection layer with a core area for glucose detection, and an external lens providing protection with tear entry and detection holes. Tears are guided to the detection area via microfluidic channels, with two detection zones used for actual measurement and verification to ensure accuracy. Enzymes and chemicals are fixed on filter paper and react with glucose in the tears to produce a color change. Compared to traditional contact lenses requiring cleanroom manufacturing, this method does not need complex manufacturing equipment, offering better adaptability. The contact lens can detect tear glucose concentrations as low as 2 mM in approximately 10 s and is advantageous due to its simplicity, mass production capability, and high sensitivity, making it suitable for glucose monitoring in diabetic patients.

Another method integrates electrodes onto paper-based microfluidic devices to detect glucose concentrations by measuring the current changes resulting from glucose oxidation reactions. This method significantly enhances detection sensitivity and quantification capabilities. Cao et al. [159] developed a 3D paper-based glucose biosensor utilizing nanomaterials and paper-based microfluidic technology, primarily employing screen-printed electrodes (SPEs) for glucose detection (Figure 6b). The study synthesized rGO-TEPA/PB nanocomposites via in situ reduction and modified them onto paper-based electrochemical sensors to improve detection sensitivity and selectivity. The sensor demonstrated rapid response and high recovery rates (81.2–107.5%) when detecting glucose in sweat, and exhibited long-term stability. The sensor offers advantages of rapid response, portability, and low cost, providing an effective solution for non-invasive health monitoring.

#### 3.1.3. 3D-Printed Microfluidics

The term 3D-printed microfluidics refers to the fabrication of microfluidic chips or devices for the manipulation and handling of small liquid volumes using three-dimensional printing technology. Compared to traditional microfluidic fabrication methods, 3D printing offers several advantages, including ease of operation, reduced manufacturing costs, greater design flexibility, and accelerated production speed. Furthermore, the integration of 3D printing with Internet of Things (IoT) devices enables remote monitoring and control of microfluidic systems [160]. Common methods used in 3D printing of microfluidics include fused deposition modeling (FDM), inkjet printing, and stereolithography. However, the resolution and precision of 3D printing generally fall short compared to traditional fabrication methods, making it challenging to achieve fine micron-scale structures. Additionally, there are limited materials suitable for microfluidics, with many materials lacking biocompatibility, optical transparency, and ideal mechanical properties [161]. This imposes higher demands on material selection.

**Figure 7:**
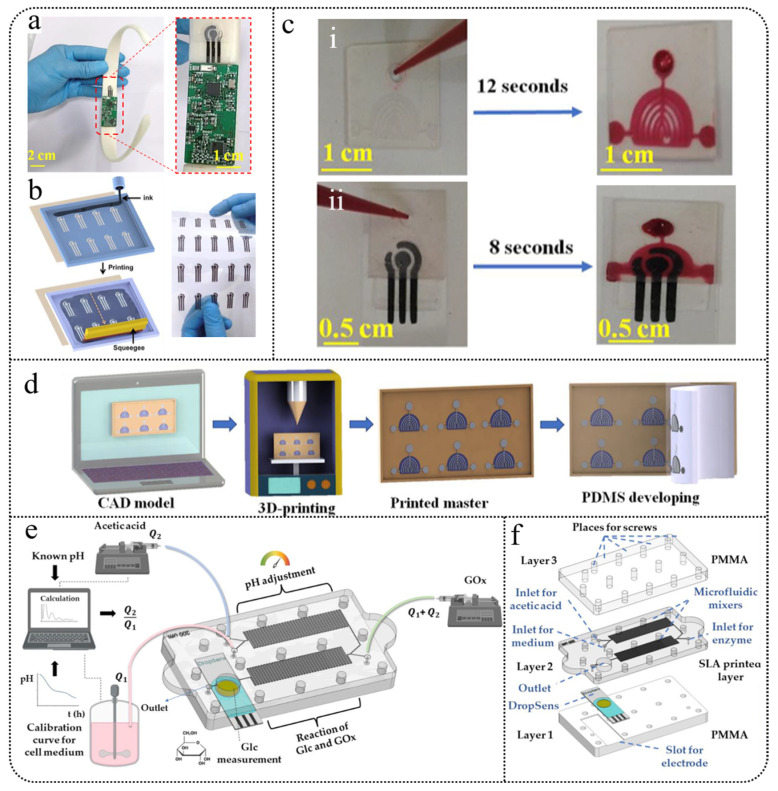
(**a**) Photograph of the neckband biosensor [163]. (**b**) Schematic and photograph of the electrode printing process [163]. (**c**) Flow of red dye in the microfluidic channels with (**ii**) and without (**i**) printed electrodes. (**d**) Schematic diagram of 3D-printed microfluidic channel fabrication [163]. (**e**) Schematic of glucose detection [164]. (**f**) Multi-layer structure of the device [164].

Furthermore, 3D-printed microfluidic technology is commonly used for glucose detection in sweat. One innovative three-dimensional printed flexible and wearable health monitor employs a continuous one-step fabrication process, integrating self-supporting microfluidic channels and single-atom catalysts. This design allows for real-time measurement of sweat rate, glucose, lactate, and uric acid concentrations, while utilizing direct ink writing technology to avoid the challenges of support material removal found in traditional methods, and addressing issues of contamination and sweat evaporation [162]. Validation studies on human skin demonstrate that this monitor provides high sensitivity and accuracy during exercise and shows improved stability compared to previous commercial enzyme-based monitors. Additionally, Mwaurah et al. [163] developed a low-cost, disposable 3D-printed microfluidic sensor designed for non-invasive, real-time blood glucose monitoring. This sensor integrates carbon-based flexible electrodes, PDMS microfluidic channels, a 3D-printed neck strap, and a Bluetooth-enabled circuit board, allowing for real-time monitoring of glucose, lactate, and uric acid levels in sweat (Figure 7a–d). By using Pd-Pt-modified reduced graphene oxide as a catalyst and performing amperometric measurements at 0.2 V, the sensor exhibits excellent stability, selectivity, and sensitivity. The microfluidic channel design enables pump-free autonomous capture and continuous sampling of sweat. Experimental results indicate that the sensor effectively measures glucose levels in sweat during fasting and postprandial phases, showing good correlation with commercial glucose meters and standard HPLC methods. This research validates the effectiveness of the neck strap-integrated microfluidic sensor for non-invasive physiological glucose monitoring, with future enhancements anticipated through the integration of artificial intelligence and smartphone applications to further improve its analytical capabilities.

In other bodily fluids, 3D-printed microfluidic technology still holds significant potential. Podunavac et al. [164] developed a novel 3D-printed microfluidic chip that integrates serpentine micromixers and electrochemical sensors, specifically designed for glucose detection in liquid samples (Figure 7e,f). The mixing performance was optimized through numerical simulations, and fluid flow rates were adjusted based on the pH of the sample. This chip demonstrates excellent performance in acetate buffer solutions and cell culture media, offering advantages such as minimal sample and reagent usage, high sensitivity, and a good linear response. It shows potential for direct online monitoring of glucose and pH changes over time in bioreactors.

### 3.2. Wearable Fabric Sensors

Wearable fabric-based electrochemical glucose sensors represent an emerging technology for real-time monitoring of glucose levels in bodily fluids. These sensors are embedded in flexible textiles, making them lightweight, comfortable, and suitable for extended wear [165]. By incorporating conductive materials and biological enzymes, such as glucose oxidase (Gox—reaction equation is as shown in Formula (5)), onto the fabric surface, these sensors leverage electrochemical principles to accurately measure glucose concentrations in sweat or other bodily fluids. Compared to traditional blood glucose testing methods, these sensors offer a more convenient and non-invasive monitoring approach, allowing users to manage their health more easily in daily life and thereby enhancing their quality of life.

(5) $$\mathrm{Glucose}+O_{2}\overset{GOx}{\to }\;\mathrm{Gluconolactone}+H_{2}O_{2}$$

**Figure 8:**
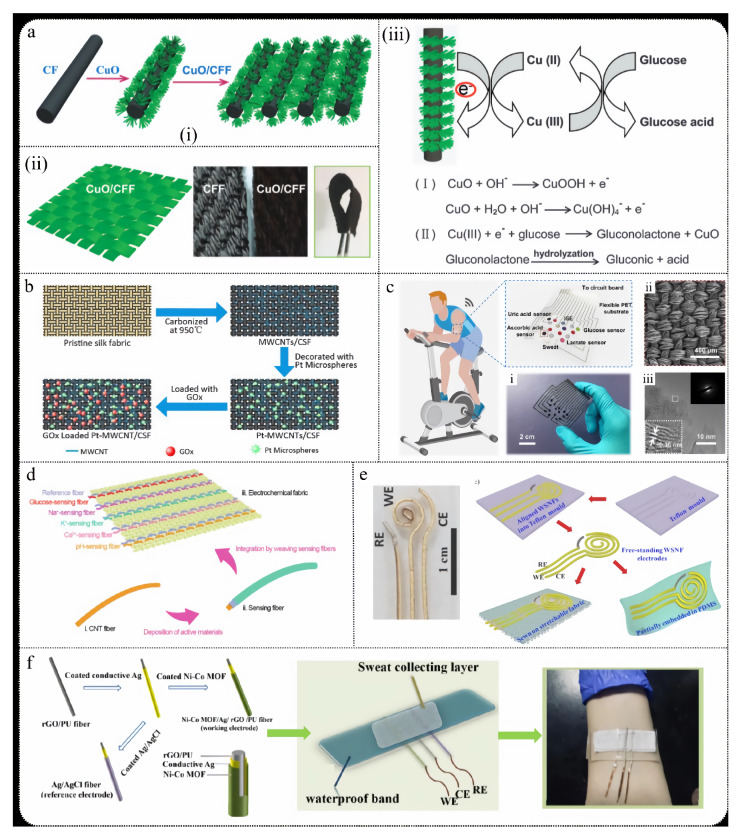
(**a**) (**i**) Preparation process of nanorod-assembled flower-like CuO composite material and (**ii**) planar view of CuO-CCF composite material. (**iii**) Mechanism of direct electrooxidation of glucose to gluconic acid on CuO/CFF electrode surface in alkaline medium [166]. (**b**) Preparation process of MWCNTs/CSF glucose sensor [167]. (**c**) Wearable sweat analysis patch: (**i**) photo of the patch, (**ii**) SEM image, (**iii**) TEM image [168]. (**d**) Electrochemical fabric with multimodal sensing capabilities [169]. (**e**) Schematic of the manufacturing process for fully enclosed WSNF electrodes (CE, WE, and RE) [170]. (**f**) Integration of NCGP glucose sensor into elastic fabric [171].

The growth of metal oxide nanoparticles on fabric fibers for non-enzymatic glucose sensing offers significant advantages. Firstly, this approach provides a larger electroactive surface area, which improves electron transfer rates, enhancing the sensor’s sensitivity and detection speed. Secondly, it addresses the stability and longevity issues associated with traditional enzymes, thereby extending the sensor’s lifespan and durability. Additionally, these sensors maintain the fabric’s flexibility and comfort, making them suitable for wearable devices. The manufacturing process is straightforward and cost-effective, which contributes to the sensor’s excellent performance in real-time health monitoring. Xu et al. [166] developed a novel ultra-sensitive glucose biosensor by growing nanorod-structured CuO clusters on carbon fiber fabric (CFF) using a simple, rapid, and environmentally friendly hydrothermal method (Figure 8a). The CuO/CFF electrode exhibits high electrocatalytic activity at 0.45 V, with a sensitivity of 6476.0 mA/mM/cm^2^, a detection limit as low as 0.27 mM, and a response time of just 1.3 s. The sensor demonstrates excellent reproducibility (R.S.D. 1.53%), long-term stability (with only 9.9% sensitivity loss over one month), and strong interference resistance. The reaction equation is as follows:

In alkaline media (common for noble metals):

Glucose + OH → Gluconate + HO + 2e^−^ (6)

(This occurs at ~0.3–0.5 V vs. Ag/AgCl on Pt/Au.)

Nickel/cobalt-based catalysts:

Ni(OH)_2_ + OH → NiOOH + H_2_О + e^−^ (7)

NiOOH + Glucose → Ni(OH),+ Gluconate(8)

As flexible electronics garner increasing attention, fabric-based electrochemical glucose sensors are also transitioning towards fiber-based approaches. Silk fabrics offer excellent flexibility and comfort, large specific surface area, high stability, ease of integration, and low cost, making them suitable for continuous glucose monitoring applications. Chen et al. [167] developed a highly conductive, flexible, and stable MWCNTs/CSF material by coating multi-walled carbon nanotubes (MWCNTs) on carbonized silk fabric (CSF) (Figure 8b). They further enhanced the conductivity and sensitivity to H_2_O_2_ by electrochemically depositing platinum microspheres (Pt microspheres) on the MWCNTs/CSF surface. The glucose sensor, with GOx immobilized on Pt-MWCNTs/CSF, exhibited excellent sensitivity (288.86 A/mM/cm^2^) within a linear range of 0 to 5 mM.

He et al. [168] developed a flexible sweat analysis patch based on silk-derived carbon textiles (SilkNCTs), which allows simultaneous detection of six health-related biomarkers: glucose, lactate, ascorbic acid, uric acid, Na^+^, and K^+^ (Figure 8c). SilkNCT features N-doped graphite structures and a layered porous architecture, providing excellent conductivity and a rich array of active sites. The patch demonstrates high sensitivity (detection limit as low as 0.27 mM), high selectivity, long-term stability (with minimal changes in electrochemical response over 4 weeks), good reproducibility, and real-time monitoring capability. Additionally, the patch integrates signal acquisition and transmission components, enabling efficient health monitoring in complex environments and showcasing broad application potential.

Electrochemical fabrics woven with sensing fiber units exhibit high flexibility and excellent breathability, maintaining structural integrity and detection capability under repeated bending and twisting. This makes them well-suited for real-time monitoring of human health. Wang et al. [169] developed sensing fibers using CNT fibers combined with various sensing materials, such as Prussian blue and GOx, and wove them into a fabric. Ag/AgCl fibers were used as reference electrodes, enabling real-time monitoring of glucose, sodium ions, potassium ions, calcium ions, and pH levels (Figure 8d). This electrochemical fabric demonstrates outstanding structural and performance stability, making it suitable for large-scale manufacturing and offering new directions for the development of next-generation wearable sensors and flexible electronic devices.

Moreover, the application of hybrid fibers has expanded the scope of glucose sensing in fabrics. Compared to single-material fibers, nanofibers made from two or more materials exhibit significantly improved mechanical properties. Additionally, the interaction between metal layers on the fiber surface and the core fiber can enhance performance, such as the synergistic effect between Au and oxygen-containing functional groups in reduced graphene oxide (rGO) (Figure 8e), which promotes the dehydrogenation process during glucose oxidation [170]. Shu et al. [171] developed a high-performance fiber electrode based on Ni-Co metal–organic frameworks/silver/reduced graphene oxide/polyurethane (Ni-Co MOF/Ag/rGO/PU) (Figure 8f). This fiber electrode, known as NCGP, enables continuous monitoring of glucose levels in sweat. The NCGP fiber electrode was fabricated using an improved wet-spinning technique, with the Ni-Co MOF coating enhancing the electrode’s surface area and catalytic activity. The sensor demonstrates high sensitivity (425.9 μA/mM/cm^2^), a broad linear range (10 μM to 0.66 mM), accuracy, stability under stretching and bending, and long-term storage stability. Combined with water-absorbing fabric and fixed on a polydimethylsiloxane film substrate, this sensor provides a high-precision non-enzymatic glucose sensor for personal diabetes management and real-time biological diagnostics.

Despite the advantages of fabrics in electrochemical glucose sensing, there are several challenges that need to be addressed, including sensor durability, comfort, wash stability, and long-term stability. Sensors must endure mechanical stresses from daily wear and multiple washes while maintaining the softness and breathability of the fabric. Furthermore, the long-term stability of biological enzymes and electrode materials is crucial, especially when exposed to environmental factors such as temperature and humidity, as well as user movements. Optimizing material selection, designing flexible structures, and developing durable encapsulation technologies are key strategies to overcome these challenges.

### 3.3. Self-Powered

Self-powered technology is of significant importance in wearable electrochemical glucose sensors, as it enables the device to operate independently of external power sources, thereby enhancing the device’s portability and sustainability. Based on the types of self-powered technologies, they can be mainly classified into triboelectric nanogenerator (TENG) technology, which converts mechanical energy from the body into electrical energy using triboelectric effects and electrostatic induction; thermoelectric generator (TEG) technology, which converts the body’s natural heat (such as body temperature) into electrical energy; biofuel cell (BFC) technology, which generates electrical energy through redox reactions of chemical substances in body fluids (such as glucose); and fabric-based wearable lithium-ion batteries (FLIBs).

#### 3.3.1. TENG

The triboelectric nanogenerator (TENG) was first invented in 2012 and combines contact electrification and electrostatic induction effects to convert mechanical energy into electrical energy. It is widely used in the manufacturing of self-powered sensors and small electronic devices [172]. One type of the triboelectric effect involves charging materials after they have separated from physical contact. A TENG typically consists of at least two parts: a conductive electrode and a dielectric material. The conductive electrode collects free charges, while the dielectric material enhances charge separation efficiency and charge storage capacity. When selecting materials for TENG, it is important to consider their charge density and durability. High charge density determines the output performance of the TENG, while high durability ensures greater stability and longer service life [173]. Due to its high energy conversion efficiency, abundant material resources, simple manufacturing processes, and multifunctional integration, TENG is regarded as a promising candidate for next-generation energy harvesting and self-powered sensing platforms.

The intelligent sensing platforms based on triboelectric nanogenerators (TENGs) offer multiple advantages, including self-powering, convenience, real-time monitoring, and biocompatibility [174]. However, the detection limits of TENG-based sensors still require improvement. Additionally, the output performance of TENG-based chemical sensors is significantly constrained by the miniaturization of devices and the delamination of conductive layers under prolonged body contact [175]. To overcome the limitations of TENGs in wearable applications, there has been considerable enthusiasm for TENGs based on hydrogels due to their flexibility, biocompatibility, and self-healing capabilities, making them suitable materials for responding to external stimuli in body sensor devices. Integrating glucose-responsive hydrogels with TENGs can achieve efficient energy conversion. Enhanced enzyme activity leads to increased ionic strength, thereby improving conductivity and polarization effects; hence, higher glucose concentrations can enhance TENG output. Moreover, metal–semiconductor–metal (MS-M) structures [176] and stretchable fiber structures [177] have also been successfully applied to glucose sensing.

#### 3.3.2. TEG

Thermoelectric generators (TEGs) are efficient and renewable solid-state energy harvesting devices that convert thermal energy into electrical energy [178]. They harness body heat to generate power, which can meet the energy needs of miniaturized devices and provide stable power support for various wearable devices. TEGs are considered potential alternative energy sources for wireless sensor nodes. However, TEG performance is limited by factors such as the length of the thermal source and heat sink, as well as the contact resistance of the thermoelectric elements. Additionally, the fabrication process for flexible thermoelectric generators is relatively complex, often requiring high-temperature operations and incurring higher costs, while low thermal resistance also restricts their power generation efficiency [179].

Kim et al. [180] developed a self-powered glucose sensor that charges a supercapacitor with electrons generated from glucose oxidation reactions, eliminating the need for an external power source to measure glucose levels. This study integrates a microneedle array sensor with a TEG, continuously powering the sensor with body heat, achieving a truly continuous and uninterrupted power supply. Experiments demonstrate that the TEG can convert body temperature differences into electrical energy, providing sufficient power support. Meanwhile, a wave-structured radiative heat sink enhances energy collection efficiency through radiation and convection cooling. This technology offers continuous and stable energy support for long-term monitoring and treatment with wearable medical devices, showcasing its potential in chronic disease management.

#### 3.3.3. BFC

With the growing emphasis on sustainability, clean energy derived from body fluids as biofuels has garnered significant attention from researchers [181]. This is primarily due to the presence of substances in body fluids, such as glucose and lactate, which can be converted into energy, making it a promising approach for powering wearable devices [182,183,184,185]. Glucose biofuel cells offer cost-effectiveness, high reproducibility, and excellent stability [186]. When combined with microfluidic devices, these cells can achieve constant, passive absorption and flow of body fluids [187], while also serving as filters to reduce interference from skin debris [188]. Additionally, microfluidic-based fuel cells have advantages in high surface area, ease of integration, and portability [189]. The integration of microfluidics with biological catalysts (such as enzymes) presents a promising outlook for energy generation from body fluids [190].

Recent reports have demonstrated the use of biofuel cells powered by tears to supply energy for smart contact lenses (SCLs). Non-biological glucose fuel cells and magnesium/air batteries can be used in combination as alternative power sources for SCLs [191]. The fuel cells derive energy from tear metabolites, while magnesium/air batteries provide power through the oxidation of a sacrificial anode (Figure 8a), generating 40 μW and 2 μW of electrical power, respectively, and lasting over 24 h. However, limitations imposed by conventional contact lens materials significantly impede mass transfer, resulting in a 82.5% reduction in power (only 7 μW). To enhance the power of biofuel cells, Yun et al. [192] utilized copper hexacyanoferrate (CuHCFe) and glucose oxidase (GOx) materials as cathodes and polypyrrole (PPy) as an anode, integrating these electrodes into the SCL to function as a battery. When immersed in tears, this setup achieves charging through the oxidation of glucose (Figure 8b). Compared to the study by Frei et al. [191], this fuel cell demonstrates a maximum discharge power more than 100 times greater (201 μW) and exhibits long-term stability. Additionally, it can be routinely charged via an external power source, overcoming the limitations imposed by contact lens materials and offering greater practicality and applicability.

#### 3.3.4. FLIB

Textiles inherently possess wearability advantages, and electronic textiles (E-Textiles), which combine electronic devices with fabrics, enable convenient and efficient monitoring of human biological signals and molecules [193]. With advancements in the Internet of Things (IoT), virtual/augmented reality, and robotics, E-Textiles can also elevate human interactions to new levels [194]. From a sensing perspective, E-Textiles are fabrics capable of performing electronic functions such as sensing, computing, displaying, and communication [195]. From an energy perspective, fiber-shaped generators and batteries generate energy while retaining the flexibility of textiles [196], laying a solid foundation for developing wearable, rechargeable devices. However, a key challenge in the development of electronic textiles is how to integrate rigid semiconductor-based circuits or other devices into the textiles to achieve effective circuit connections [197]. This challenge requires ensuring that circuits maintain sufficient flexibility, stability, and environmental durability while enduring pressure and strain, as well as ensuring the reliability and performance of the circuits during material transitions.

In 2021, a study on woven lithium-ion batteries was featured in *Nature* [198]. This research broke traditional charging paradigms by using fibers as a new form of batteries, enabling charging of smartphones through textile integration. This innovation is revolutionary, suggesting that energy supply can be achieved without relying on traditional batteries, and it offers greater convenience, compactness, and flexibility, significantly expanding the application scenarios of fiber batteries. Building on this technology, integrating it with wearable electrochemical fabrics not only saves substantial space but also enhances comfort, providing a significant improvement for future wearable glucose monitoring devices.

Recently, the research team has developed a high-performance fiber battery with a polymer gel electrolyte [199]. This design incorporates aligned and networked electrode fibers to ensure more stable contact between the polymer gel electrolyte and the electrodes, resulting in lower internal resistance and higher discharge capacity. When scaled up to garment size, this battery can safely power 20 smartphones from 0% to 100%. Additionally, the fiber battery maintains its functionality under extreme temperature conditions and exhibits excellent energy storage performance.

## 4. Minimally Invasive Technology

Minimally invasive technology refers to methods used in medical or biological testing processes that aim to minimize tissue damage or disruption, thereby reducing patient pain, trauma, and recovery time. Compared to traditional invasive surgeries or testing techniques, minimally invasive technology typically utilizes smaller instruments or devices, such as microneedles, subcutaneous implants, or localized punctures, to avoid extensive tissue cutting or damage.

### 4.1. Microneedles

Due to the pain and discomfort caused by conventional blood collection methods, diabetes patients often have poor adherence to glucose monitoring. Microneedles (MNs), with their minimally invasive and painless advantages, can penetrate the skin’s stratum corneum to reach the ISF in the dermis, allowing glucose extraction without affecting nerve endings, thus reducing pain. This makes them widely applicable in wearable electrochemical glucose sensors. Microneedles are typically made from biocompatible materials, playing a crucial role in enhancing the sensor’s comfort, portability, long-term wearability, and accuracy.

MNs were initially developed for painless drug delivery [200,201,202,203], and their application has since been extended to glucose monitoring [204]. Due to the high correlation between glucose in ISF and blood glucose levels, MNs provide a minimally invasive, convenient, and accurate method for glucose monitoring by enabling ISF collection and glucose detection. The primary mechanisms for ISF collection include hydrogel microneedle diffusion and absorption, capillary action in porous and hollow microneedles, osmotic pressure gradients, and external pressure driving [205]. These methods lay a solid foundation for efficient ISF collection and continuous glucose monitoring.

Early MNs had a tip radius of 15–40 μm and needed to penetrate the skin 700–1500 μm to reach the dermal layer, collecting 1–10 μL of ISF for glucose level measurement [206,207]. By improving the materials used in MNs, higher surface areas can be achieved, enabling the detection of multiple biomolecules. For example, microneedles made from epoxy resin-based negative photoresist (SU850) have a tip diameter of 35 μm, a length of about 1000 μm, a base width of 600 μm, and a spacing of 1200 μm. These microneedles can measure glucose concentrations as low as 0.5 mM with a response time of 15 s. Additionally, the MN array can be modified and allocated to working and reference electrodes, facilitating multiplexing or single-analyte sensing [208].

Patch-based MNs have rapidly advanced in glucose biosensing and continuous blood glucose control. They are not only suitable for wearable use, making them convenient for daily monitoring, but also offer extended functionalities for tracking other biomarkers. Parrilla et al. [209] introduced a highly stable electrochemical glucose biosensor using a dual-layer redox mediator structure. They developed an MN array capable of penetrating the skin to extract interstitial fluid and integrated it with a biosensor to create a microfluidic sensing patch. This patch uses an injection pump to extract ISF and provides real-time glucose monitoring (detection range: 2.5–22.5 mM), demonstrating excellent analytical performance and reversibility. The reliability of the microfluidic patch was validated through in vitro and in vivo experiments using pig skin. This technology presents potential for painless and cost-effective diabetes management.

### 4.2. Subcutaneous Sensor

A subcutaneous sensor is a device implanted beneath the skin, typically within the dermis or subcutaneous fat layer, at a depth of about 1 to 5 mm. Due to its shallow implantation depth, the subcutaneous sensor is minimally invasive, usually requiring only a minor surgical procedure or needle insertion. It allows for continuous monitoring over several days to months. This sensor can monitor glucose levels in the ISF in real time, making it suitable for diabetes management, chronic disease monitoring, and exercise health tracking.

Henninger et al. [210] evaluated the biocompatibility and tissue response of a needle-type glucose sensor (NTS) implanted subcutaneously in male rats through gene expression and histological analysis, comparing it with the commercially available MiniMed continuous glucose monitoring system (CGMS). The study found that NTS elicited minimal tissue irritation and exhibited good biocompatibility. Although the MiniMed system failed to accurately detect glucose changes, it revealed acute-phase responses, providing important insights for improving glucose sensor design and application. Huang et al. [211] developed a differential dielectric sensor for subcutaneous implantation based on microelectromechanical systems (MEMS). The sensor consists of a sensing module and a reference module, which measure glucose concentrations accurately by detecting the signal differences between the two modules, thus eliminating non-specific interference. In both in vitro and in vivo tests, this sensor demonstrated higher accuracy and stability compared to single-module designs, showing excellent stability even under temperature variations. Its sensitivity covers a glucose concentration range of 0–500 mg/dL and exhibits good resistance to environmental interference, making it promising for clinical continuous glucose monitoring applications.

### 4.3. Microdialysis

Microdialysis is a technique used for in vivo or in vitro sample analysis that involves inserting a small probe or catheter into the subcutaneous or tissue areas to collect fluid samples through tiny incisions, allowing for continuous monitoring of glucose and other biomarkers [212]. Li et al. [213] proposed a high-precision microdialysis method to provide reference values for glucose concentrations in subcutaneous interstitial fluid (ISF) to evaluate the accuracy of non-invasive and minimally invasive continuous glucose monitoring systems. By optimizing parameters such as the microdialysis perfusion rate, temperature, and glucose concentration around the probe, and validating the method in simulation systems and animal experiments, the results showed that the average deviation between calibrated glucose concentrations and actual measurements was 3.7%. This study used the Jacobson recovery model to explain the relationship between microdialysis recovery, membrane surface area, and perfusion rate, providing a high-precision method for measuring interstitial glucose and demonstrating significant clinical application potential. Najmi et al. [214] proposed a non-enzymatic microfluidic glucose sensor based on Pt-Ni nanoparticles and multi-walled carbon nanotubes for routine glucose detection, using microdialysis technology to measure glucose in ISF. The sensor exhibits high sensitivity, a wide linear range, good repeatability and stability, and resistance to chloride contamination, with a compact structure and minimal dialysate consumption, making it suitable for continuous glucose monitoring in implantable devices. The effectiveness of its structure was validated through numerical simulations, providing a new solution for non-enzymatic glucose detection in microfluidic systems with significant biomedical application potential.

## 5. Glucose Monitoring Applications Based on Biological Fluids

### 5.1. Tear Sensors

Tears are secreted by the lacrimal glands and produced through the combined action of the main and accessory lacrimal glands, corneal and conjunctival epithelial cells, and meibomian glands. They contain small amounts of glucose, proteins, and electrolytes and play a role in nourishing and protecting the surface of the eye, maintaining ocular moisture and transparency [215,216,217]. When collecting tear fluid, methods such as Schirmer strips and microcapillaries are commonly used [218]. However, it is important to keep the collection tools clean to prevent cross-contamination of tears, control the collection time to avoid eye irritation and changes in tear composition, and store the collected tear fluid under dry, clean, and refrigerated conditions to ensure sample reliability.

Because tears are a micro-sample biological fluid, there is often an issue with insufficient sample volume during collection. Additionally, there are variations in tear composition between different individuals, and special populations such as infants, elderly individuals, or patients with specific eye conditions may present challenges in the tear collection process. Therefore, there is a greater need to optimize collection methods, reduce sample volume, and ensure timely analysis.

#### 5.1.1. Smart Contact Lenses (SCLs)

The electrode systems for tear-based electrochemical sensors have evolved from strip electrodes [219] to concentric ring electrodes [220] and then to wireless/resonant sensing [221], with the latter often integrated into smart contact lenses (SCLs). SCLs offer significant advantages in monitoring the eye and tear fluid, as they directly adhere to the corneal surface, providing real-time, non-invasive medical diagnostics. However, previous reports on SCLs have indicated that opaque and fragile components may obstruct the user’s vision and potentially harm the eye while implementing electronic device operations [222]. Moreover, integrating bulky and expensive devices onto SCL sensors severely impacts the user’s normal activities. Additionally, issues with powering electronic devices have significantly affected the efficiency of wireless data transmission [223]. Consequently, researchers are compelled to actively develop non-invasive, convenient, and low-energy-consuming SCL glucose monitoring systems.

**Figure 9:**
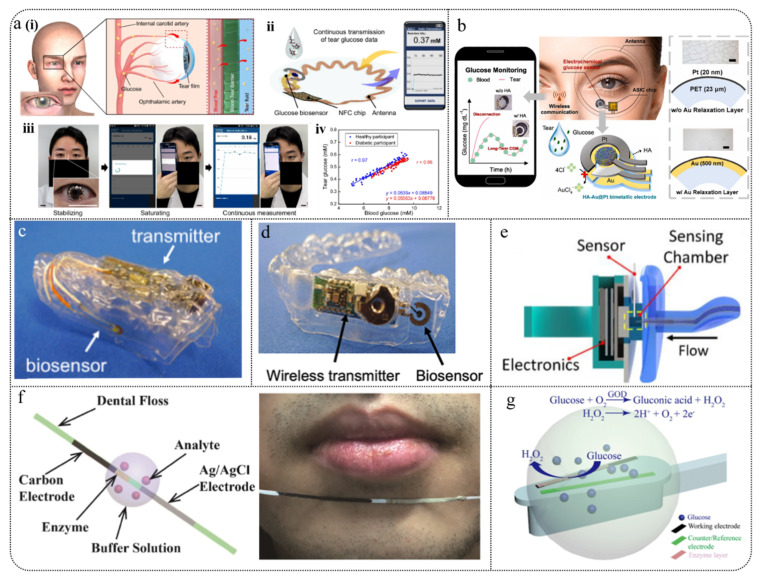
(**a**) In-depth correlation analysis of tear glucose and blood glucose using wireless smart contact lenses (SCLs): (**i**) Schematic diagram of human eye plasma leakage. (**ii**) Tear glucose monitoring system with SCL and smartphone. (**iii**) Photo of a human participant wearing the SCL and using it for tear glucose measurement. Scale bar, 2 cm. (**iv**) Correlation analysis between tear glucose and blood glucose levels [225]. (**b**) SCL based on Au@Pt bimetallic electrodes [141]. (**c**) Oral protector biosensor [236]. (**d**) MG glucose sensor [237]. (**e**) Pacifier glucose sensor [238]. (**f**) Dental floss glucose sensor [240]. (**g**) Toothbrush glucose sensor [240].

The glucose content in tear fluid is correlated with blood glucose levels, but this relationship is somewhat controversial due to significant differences in tear composition caused by various collection methods. Additionally, the lag time between tear glucose and blood glucose is a crucial factor determining their correlation [224]. The characteristic of tear glucose being measurable only once limits continuous glucose tracking, making it challenging to determine the lag time relationship between tear glucose and dynamic blood glucose levels. Smart contact lenses (SCLs) provide the capability for continuous measurement of tear glucose, offering a valuable platform to analyze the correlation between tear glucose and blood glucose. Recently, Lee et al. [225] proposed a new method to analyze the precise correlation between tear glucose and blood glucose. The researchers used SCLs to determine whether reflex tearing was induced in individuals, establishing a “personalized lag time” model. This model enabled the precise identification of each individual’s lag time during prolonged continuous glucose measurements (Figure 9a). Testing with 20 participants over three days showed Pearson correlation coefficients of 0.97 for healthy individuals and 0.95 for diabetic patients, demonstrating a positive correlation between tear glucose and blood glucose.

Using bimetallic electrodes in tear-based electrochemical glucose sensors within smart contact lenses (SCLs) offers significant advantages. The synergistic effect of bimetallic materials can greatly enhance catalytic activity and sensitivity, providing faster response times and a wider operational voltage range. Additionally, bimetallic electrodes exhibit higher stability and durability in the complex tear fluid environment, making them suitable for long-term monitoring. These benefits make bimetallic electrodes an ideal choice for glucose sensors in smart contact lenses. Lin et al. [226] developed a continuous glucose monitoring (CGM) SCL sensor using hyaluronic acid (HA)-modified Au@Pt bimetallic electrodes. The glucose levels measured showed a high correlation with commercial glucose meters (ρ = 0.88) and clinical accuracy (98.6%) (Figure 9b). Due to the lower ductility of Pt and the tendency of Au to dissolve in chloride-containing solutions (tears contain 118–138 mM of chloride ions), the Au@Pt electrodes effectively combine the corrosion resistance of Pt with the ductility of Au. As a result, these electrodes exhibit high stability. The sensor’s sensitivity reaches 110.92 μA/mM/cm^2^, which is over ten times higher compared to the study by Zou et al. [227].

Moreover, by integrating self-powered technology with circuit integration, the application range of smart contact lenses (SCLs) has been significantly expanded. Lin et al. [228] developed a self-powered flexible organic electrochemical transistor (OECT) sensor for real-time monitoring of glucose and calcium ions in tears. This sensor demonstrated a semi-logarithmic response to glucose and calcium ions under conventional LED light, enabling wireless data transmission to a laptop. Experimental results indicated that the device could detect glucose concentrations of 0.74 ± 0.03 mM and up to 0.97 ± 0.05 mM after meals, which aligns with the concentration ranges found in normal and diabetic individuals in the literature. Additionally, the sensor has a response time of approximately 1.3 s, a sensitivity of about −22.72%/mM, and a detection limit of 12.57 μM. Despite some instability of the signal on flexible substrates, the overall performance still demonstrates good monitoring capabilities and application potential.

A novel soft smart contact lens has been introduced, integrating glucose sensors, wireless circuits, and display pixels for real-time, non-invasive medical monitoring [224]. This lens uses transparent and stretchable nanomaterials to avoid common issues of visual obstruction and discomfort found in traditional devices. The sensor detects glucose in tears using graphene channels and glucose oxidase (GOx), with catalase (CAT) enhancing sensitivity. The device features a rapid response (about 1.3 s), high sensitivity (−22.72%/mM), and a low minimum detection concentration (12.57 μM). Additionally, the sensor exhibits good selectivity for glucose, long-term stability, and mechanical durability. The smart contact lens displays glucose levels in real time via LED pixels and automatically turns off the LED when glucose concentrations exceed 0.9 mM, providing intuitive high-glucose indication. This smart contact lens offers a new solution for non-invasive in-body testing while maintaining high transparency and real-time glucose monitoring and data display capabilities.

#### 5.1.2. Ocular Disease Detection

Diabetes, although primarily a disorder of insulin metabolism caused by elevated blood glucose levels, has significant systemic impacts and can lead to a range of complications, including ocular diseases. Common eye conditions associated with diabetes include diabetic retinopathy (DR) [229], diabetic cataract [230], and dry eye syndrome (DES) [231]. In healthy individuals versus patients with diabetes and DR, the glycosylation patterns of tear proteins are highly conserved between individuals, with minor changes in glycosylation observed in the tears of diabetic and DR patients. However, certain O-glycans exhibit significant variations in patients [232]. Cullen et al. [233] compared the tear fluid, corneal sensitivity, and conjunctival tissue characteristics among diabetic cataract, non-diabetic cataract, and non-diabetic non-cataract dogs. The study found that tear secretion, corneal contact threshold, and tear film break-up time were significantly lower in diabetic cataract dogs compared to the other two groups, and the glucose concentration in their tears was also notably higher. A non-invasive ocular front sensor system holds significant application value in simultaneously monitoring both diabetes and DES [231]. Researchers developed a sensor system using multiple sensors to collect and transmit tear fluid with minimal contact to the ocular surface, requiring only 0.6 to 1.0 μL of tear fluid without causing damage to the eye tissue. Human trials indicated that the sensor system showed high correlation with clinical blood glucose meters in detecting glucose levels, and it demonstrated high sensitivity (91.7%) and specificity (83.3%) for diagnosing DES conditions.

### 5.2. Saliva Sensor

Saliva is secreted by the salivary glands and plays a crucial role in digestion, lubrication, antimicrobial activity, oral hygiene, and maintaining pH balance. Research has shown that glucose concentration in saliva varies across different tooth surfaces, with higher glucose levels found on the upper and lower incisors compared to other surfaces, and higher levels on the buccal surfaces of lower molars compared to the lingual surfaces, which have the lowest concentration [234]. Saliva can be collected on-site without special equipment or facilities, making it suitable for rapid collection and testing scenarios. However, during saliva collection, maintaining oral hygiene is important; excessive stimulation of oral mucosa should be avoided to prevent discomfort or damage; care must be taken to avoid contamination of saliva samples during storage and transport to ensure accuracy in subsequent analysis; and temperature and humidity conditions should be controlled to prevent sample degradation or damage.

The concept of the “Cavitas sensor” was first introduced in 2015 [235]. This innovative medical sensor, positioned between implanted and wearable sensors, allows for non-invasive glucose monitoring in various body cavities, such as the oral cavity, pharynx, and conjunctival sac [236] (Figure 9c). The sensor not only enables real-time, continuous, and wireless measurement of glucose in saliva but also provides protection to the oral cavity. Its electrode system comprises Pt and Ag/AgCl, with glucose oxidase (GOx) immobilized on a custom-made oral guard frame via Poly(MPCco-EHMA) (PMEH) capture. The sensor performs remote glucose measurement through a wireless transmitter and is capable of detecting glucose levels ranging from 5 to 1000 µM, with over 5 h of extended real-time monitoring.

Other substances in the oral cavity, such as ascorbic acid (AA) and uric acid (UA), can interfere with glucose measurement results. Additionally, saliva collection can be challenged by sample contamination. To address these issues, researchers have developed a dental guard (MG) glucose sensor [237] (Figure 9d) that uses interference suppression membranes by coating the electrodes with cellulose acetate (CA) and implementing oral hygiene and sample pretreatment methods. This sensor can connect to mobile applications to search for wireless measurement instruments around the MG sensor, receive data, and plot and store it. This innovation eliminates the need for USB-connected BLE receivers in traditional wireless measurement systems, thereby expanding the mobility range of the MG sensor. Compared to previous research, this sensor offers a wider detection range (1.75–10,000 µM >> 5–1000 µM) and a lower detection limit (1.75 µM < 5 µM).

The development of wearable devices for infants has broadened the application range of saliva glucose sensors [238]. Researchers have integrated sampling and measurement systems into a single platform, achieving one-way saliva flow with a pump-free system and incorporating an external collection outlet on a pacifier for ease of use by infants (Figure 9e). This device combines saliva sampling with electrochemical sensing technology and integrates a miniature wireless electronic device on the pacifier platform, allowing for prompt alerts to the wearer or parents regarding abnormal glucose levels, enabling real-time and selective monitoring of glucose levels in infants or adults. Additionally, this platform can be used to monitor other biomarkers, providing multi-analyte monitoring capabilities. The linear range of the device is 0.1–1.4 mM, with a limit of detection (LOD) and a limit of quantitation (LOQ) of 0.04 mM and 0.1 mM, respectively. However, clinical applications still face challenges, including enhancing sensor stability, reducing biological contamination, and ensuring accuracy and reliability over extended periods of use.

With ongoing innovations in saliva glucose sensor technology, new sensors themed around smart toothbrushes [239] (Figure 9f) and smart dental floss [240] (Figure 9g) have emerged. The smart toothbrush integrates an amperometric biosensor, which non-invasively monitors glucose in saliva during brushing to assist diabetic patients in managing blood glucose levels. This sensor uses a two-electrode configuration: carbon graphite ink serves as the working electrode and Ag/AgCl ink as the reference/counter electrode, with glucose oxidase (GOx) immobilized on the working electrode. It has a detection range of 0.18 mM to 5.22 mM and provides results in less than 5 min. Although its performance is somewhat inferior to the Cavitas sensor and pacifier sensor, this research demonstrates the potential for integrating various electrochemical sensors into toothbrushes for specific detection of saliva or toothpaste analytes. Similarly, the smart dental floss glucose sensor employs a comparable approach, using carbon graphite and Ag/AgCl inks, with GOx immobilized on the working electrode. Compared to the smart toothbrush sensor, this dental floss sensor offers a wider detection range (0.048 to 19.5 mM). However, further research is needed to optimize its miniaturization and signal processing systems.

### 5.3. Sweat Sensor

During physical activity, muscle contractions cause an increase in body temperature. To maintain normal body temperature, the body regulates sweat production through the autonomic nervous system. When body temperature rises, the hypothalamic temperature regulation center becomes activated and sends signals via the nervous system to the sweat glands in the skin, stimulating sweat production. Real-time monitoring of glucose levels in sweat during exercise can help reduce the risk of hypoglycemia during physical activity. Since sweat is readily available and does not require external stimulation tools during exercise, developing glucose sensors based on sweat shows great potential in the field of exercise monitoring.

However, an important factor often overlooked during exercise is the dynamic fluctuations in sweat. These fluctuations can affect test results and, consequently, the accuracy of sensors, making calibration essential. Calibration methods based on pH and temperature (*T*) are related to enzyme activity [241]. Specifically, enzyme activity increases with rising temperature (*T*), while there is a negative correlation between pH and enzyme activity. Under reference conditions of pH 6.6 and T at 20 °C, a first-order calibration algorithm is applied to obtain dynamic calibration equations for the slope (*k*′) and intercept (*m*′). These equations are used to determine the dynamic glucose concentration in sweat during in vivo testing:

(9) $$k^{\prime }=\frac{{slope}_{pH}}{{slope}_{6.6}}\cdot \frac{{slope}_{T}}{{slope}_{20}}\cdot k$$

(10) $$m^{\prime }=\frac{{intercept}_{pH}}{{intercept}_{6.6}}\cdot \frac{{intercept}_{T}}{{intercept}_{20}}\cdot m$$

In the equation, *k* and *m* represent the initial in vitro calibration slope and intercept, respectively.

#### 5.3.1. Exercise-Induced Hypoglycemia

Exercise-induced hypoglycemia refers to a physiological condition where blood glucose levels fall below the normal range during exercise. It typically occurs in the later stages of exercise or after prolonged, high-intensity physical activity, due to significant glucose consumption by the muscles and relatively insufficient insulin and glycogen supply. Anticipating and detecting exercise-induced hypoglycemia in advance is crucial.

**Figure 10:**
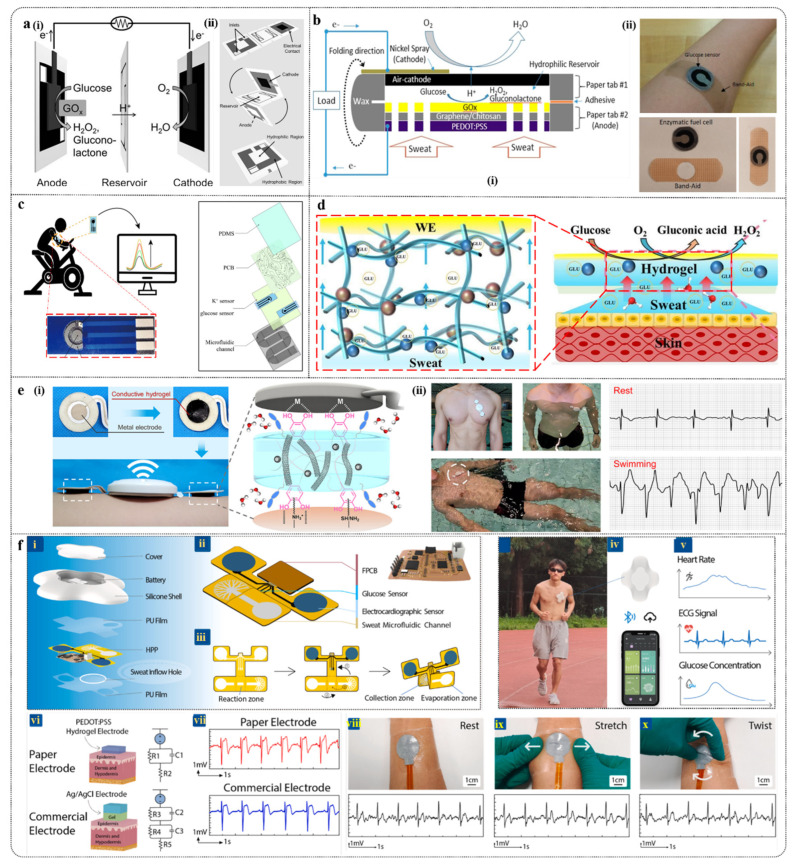
(**a**) Enzyme fuel cell-based glucose sensor: (**i**) principle; (**ii**) assembly process [242]. (**b**) (**i**) Sensor reaction mechanism; (**ii**) disposable patch sensor attached to the arm [243]. (**c**) Device structure diagram [244]. (**d**) PVA/AuNPs@PDA self-healing hydrogel detection principle [246]. (**e**) (**i**) Schematic of wet gel hydrogel-integrated bioelectronic device; (**ii**) ECG signals recorded before and during swimming [245]. (**f**) High-porosity PEDOT:PSS hydrogel self-assembled on paper fibers: (**i**) diagram of the HPP system; (**ii**) different parts of the device; (**iii**) origami method; (**iv**) Custom-developed HPP device for electrophysiological (ECG) and biochemical (glucose) monitoring. Schematic illustration of the device and the signal transmission through the Bluetooth chip to a cell phone APP; (**v**) Schematic illustration of HPP motion test results; (**vi**) equivalent circuit of the electrode; (**vii**) comparison of ECG signals; (**viii**) resting ECG; (ix) stretched ECG; (x) twisted ECG [247].

Glucose/oxygen enzyme fuel cells can provide power for sensors. Early reports on this type of fuel cell for glucose monitoring [242] described the use of chitosan/graphene oxide anodes to facilitate stable current generation. The sensor showed a linear response in the glucose range of 1 to 5 mM (R^2^ = 0.996), with a sensitivity of 0.02 µA/mM (Figure 10a). Although this sensor has a very low cost (approximately USD 0.15) and can generate sufficient current and power to sensitively monitor high glucose concentrations in blood (~1 mg/mL), it cannot detect glucose concentrations lower than 0.2 mg/mL in sweat. To further optimize the sensor’s detection limit, researchers developed a disposable glucose sensor on paper by combining electronic and microfluidic structures (Figure 10b), using materials such as graphene/chitosan/PEDOT:PSS [243]. This sensor can adsorb sweat via capillary action and generate electrochemical signals, enabling direct electron transfer between GOx and the anode and real-time glucose detection without an external power source. The improved device can detect glucose concentrations below 0.2 mg/mL (1109 µM) with a linear range of 0.02 to 1.0 mg/mL (111 to 5548 µM) (R^2^ = 0.989) and a significantly improved sensitivity of 1.35 µA/mM. It not only features low cost and ease of use but also demonstrates potential applications in diabetes management. Furthermore, integrating a PCB circuit board with glucose and K+ ion sensors and using microfluidic channels for sweat collection allows for dehydration and hypoglycemia alerts by setting thresholds for K+ and glucose concentrations. Alerts can be triggered when K+ concentrations fall below 7.5 mM and glucose concentrations are below 60 µM and 120 µM [244]. This glucose sensor demonstrates a lower detection limit, triggering an alarm when concentrations fall below 60 µM (Figure 10c). This significantly aids in providing alerts for impending dehydration and hypoglycemia during exercise, allowing for timely hydration and rest.

#### 5.3.2. Glucose and ECG

Electrocardiogram (ECG) plays a crucial role in exercise monitoring. By tracking ECG during exercise, it is possible to monitor heart rate variations and detect arrhythmias, such as tachycardia, bradycardia, and atrial fibrillation. Heart rate variability is a key indicator for assessing exercise load and cardiovascular adaptation, while timely detection of arrhythmias helps reduce cardiac risk. Combining ECG with glucose electrochemical monitoring platforms allows for a comprehensive assessment of both cardiovascular health and glucose levels through sweat generated during exercise. This integrated approach not only conserves resources but also extends the range of exercise monitoring applications, offering significant value in healthcare and fitness management.

Hydrogels have demonstrated significant potential as flexible/wearable sensors for detecting various external stimuli, including monitoring human movement and glucose levels in sweat. The synergistic effects between hydrophobic components and catechol components in mussel foot proteins endow them with adhesive properties. Mussel-inspired chemical methods can impart self-healing and adhesive characteristics to hydrogels. Using polydopamine (PDA), polyacrylamide (PAM), and ethyl acrylate (C2) as primary materials, the PDA-modified conductive nanomaterials are well-dispersed within the hydrogel, forming stable conductive pathways. Consequently, PDA-functionalized CNT (pCNT) serves as a beneficial nanofiller, endowing the PDA-PAM-C2 hydrogel with conductivity [245]. This hydrogel can adhere on its own in humid environments and exhibits good conductivity and flexibility. When integrated into a wearable wireless device, it can acquire real-time ECG signals during underwater activities and wirelessly transmit the signals to a smartphone application (Figure 10e(i)). The hydrogel replaces two traditional disposable gel electrodes and adheres to the left chest via hydrogen bonding, cation–π interactions, and covalent bonds. According to the ECG data, the signals collected in both resting and swimming states are stable, with the dynamic ECG waveforms showing higher amplitudes compared to those at rest (Figure 10e(ii)). However, the team did not explore the application of this technology for glucose monitoring, which is a missed opportunity.

Meanwhile, another team, also inspired by mussels, developed a self-healing glucose sensor by incorporating gold nanoparticles@polydopamine (AuNPs@PDA) into a polyvinyl alcohol (PVA) hydrogel matrix. The PVA/AuNPs@PDA self-healing hydrogel, obtained through cyclic freeze–thaw processes, enables specific glucose detection [246]. Figure 10d illustrates the hydrogel preparation process and detection principle. The glucose concentration in human sweat was measured using a miniature electrochemical workstation circuit. Compared to studies by Fischer [242], Cho [243], and Ma [244], this hydrogel significantly improves the detection limit by three orders of magnitude (0.9 μM << 60 μM << 1109 μM) and has a much narrower detection range (1.0 μM ~ 200.0 μM << 111 ~ 5548 μM). Therefore, developing wearable devices suitable for both ECG and glucose monitoring using mussel-inspired chemistry holds great promise for the future.

Additionally, high-porosity PEDOT:PSS hydrogels self-assembled on paper fibers enable simultaneous monitoring of low-impedance ECG and glucose levels [247]. PEDOT:PSS provides excellent conductivity and hydrophilic wetting for the hydrogel–paper patch (HPP), facilitating efficient electron transport and rapid diffusion of substances. Platinum (Pt) nanoparticles were electrodeposited on the HPP surface using a constant-potential method, and GOx was immobilized, achieving a sensor sensitivity of 325.99 ± 0.8 μA/mM/cm^2^ with a limit of detection (LOD) as low as 10.3 μM. The sensor responds to glucose concentrations within the range of 0 to 12 mM, outperforming most glucose sensors. Furthermore, by integrating the HPP with a flexible printed circuit board (FPCB) and placing it on the chest, it enables monitoring of ECG and glucose levels during physical activity. Stretching and twisting tests on the wrist show clear P waves and QRS complexes in the ECG, indicating that the HPP is resistant to interference caused by electrode deformation (Figure 10f).

### 5.4. Urine Sensor

Urine is a biological fluid formed through multiple steps including filtration, reabsorption, and secretion in the kidneys and is ultimately expelled from the body via the urethra. It plays a crucial role in maintaining the body’s water balance, electrolytes, acid–base balance, blood pressure regulation, and detoxification. When glucose levels in the urine exceed 0.8 mmol/L, it indicates high blood glucose, typically associated with diabetes [248]. The glucose concentration in urine can be influenced by factors such as diet and fluid intake and has relatively low sensitivity, making it less accurate for reflecting short-term blood glucose fluctuations. However, the simplicity, non-invasiveness, and traceability of urine collection offer a convenient alternative to blood glucose testing.

With appropriate chemical engineering treatment, nanoparticles exhibit enzyme-like catalytic activity, known as “nanozymes,” providing a rich resource of nanomaterials for various applications [249]. These nanoparticles demonstrate diverse shapes, high porosity, and chemical stability. In the study of glucose determination in urine, metal nanoparticles are often used as non-enzymatic sensors for glucose detection. Hydrothermal synthesis of Ni and Co hydroxides followed by calcination in air yields nickel–cobalt spinel (NiCo_2_O_4_) nanospheres, which can be used to modify electrochemical non-enzymatic microsensors for accurate glucose detection in urine [250]. An alarm circuit can detect glucose concentrations in urine exceeding a set threshold and issue an alert signal, exhibiting high sensitivity (3449.14 μA/mM/cm^2^), good linearity (1 μM to 100 mM), and a low detection limit (0.376 μM) as well as repeatability and long-term stability. However, various interfering substances present in urine, such as other carbohydrates and drug residues, can affect the accuracy of glucose detection. Additionally, contamination during urine collection remains a significant challenge.

**Figure 11:**
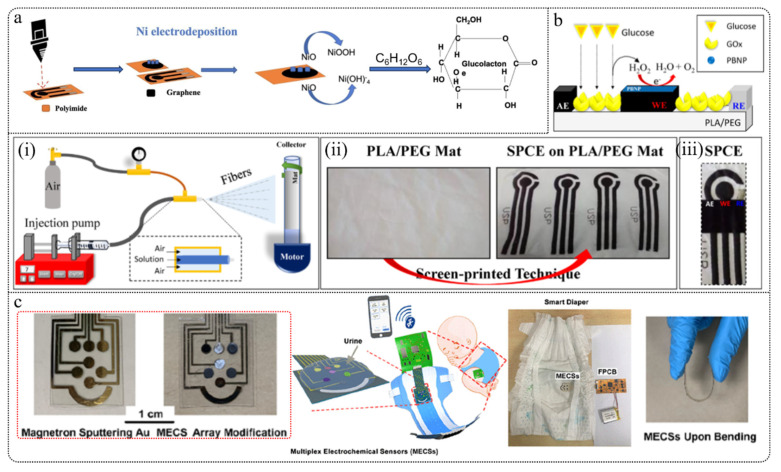
(**a**) Urine glucose monitoring strategy based on LIG [248]; (**b**) urine glucose monitoring strategy using biodegradable materials: (**i**) Illustration of the experimental setup to produce PLA/PEG mats with SB-spinning. (**ii**) Picture of PLA/PEG mat after spinning on the left, and PLA/PEG mat containing four screen-printed devices on the right. (**iii**) Picture of the SPCE containing auxiliary, working, and pseudoreference electrodes made on a PLA/PEG mat as the substrate. [251]; (**c**) glucose monitoring strategy based on diapers [252].

Laser-induced graphene (LIG) modified with nickel (Ni) nanoparticles as an electrocatalyst can address issues related to selectivity and surface contamination (Figure 11a) [248]. A urine glucose sensor prepared using the potential amperometry method exhibits rapid response time (<3 s), high sensitivity (5796.18 μA/mM/cm^2^), wide linear range (12 μM to 1.5 mM), and low detection limit (0.0152 μM). This represents an improvement of over 20 times in detection limit compared to nickel–cobalt spinel (NiCo_2_O_4_) nanosphere-modified electrochemical sensors. The sensor demonstrates high reliability in glucose detection, excellent interference resistance, and long lifespan, making it suitable for wearable sensors detecting glucose across different concentration ranges. However, long-term use may be affected by environmental conditions, potentially leading to decreased sensor performance or failure. Additionally, efforts are needed to reduce manufacturing costs and simplify the operational process.

Low-cost, environmentally friendly, and biodegradable materials align with sustainable development principles. They have minimal environmental impact and are inexpensive and easy to fabricate, which promotes the widespread adoption of glucose detection technologies. A new electrochemical biosensor architecture uses polylactic acid (PLA) and polyethylene glycol (PEG) fiber mats as substrates. Carbon electrodes are screen-printed and directly connected to glucose oxidase (GOx), enabling electrochemical glucose detection at low potentials (Figure 11b) [251]. This biosensor can detect glucose in synthetic urine within a linear response range of 0.5 to 5.5 mM and operates stably for at least 60 days, with each biosensor unit costing less than USD 0.25. When measuring glucose in human urine samples, the sensor performs at a level comparable to standard methods.

With the continuous advancement of wearable technology, diaper-form wearable glucose sensors have pioneering significance for real-time monitoring of metabolites like glucose in urine [252]. A self-powered glucose biosensor made from cellulose fiber textiles exhibits excellent bending and stretching properties. When combined with a diaper, it shows good linear response and low detection limit (3.95 µM) within a glucose concentration range of 0 to 50 mM (Figure 11c). The sensor also demonstrates good selectivity, reproducibility, and long-term storage stability, maintaining stability across different pH levels and achieving a recovery rate of 95.6% to 105.9% in synthetic urine. Compared to reusable diapers, disposable ones are more practical due to their lower cost. Future developments could focus on designing separable sensor layers and diaper layers, utilizing inexpensive diapers for single-use urine monitoring, which offers greater convenience and potential in care applications.

### 5.5. ISF Sensor

ISF is a physiological fluid that fills the intercellular spaces, containing a certain amount of glucose and participating in cellular metabolism. ISF may provide a more accurate indicator than blood because it is less prone to clotting and is highly sensitive to local tissue changes. Existing minimally invasive sampling methods typically use microneedles (MNs) [253], which transport ISF to the outside using appropriate driving forces. Additionally, needle-free sampling methods [254] and reverse iontophoresis [255,256] have also been developed. However, there are currently few products on the market for ISF glucose sensors based on MNs. Echo Therapeutics is developing a non-invasive, wireless CGM system. The developed needle-free skin preparation device is a platform technology with long-term potential, enhancing skin permeability to extract analytes such as glucose. Integrity Applications is focused on breakthrough innovations in non-invasive blood glucose monitoring devices, currently developing non-invasive technologies using ultrasound, heat, and electromagnetic methods, combined with proprietary algorithms for weighted glucose measurements. Table 2 summarizes recent advancements in MN ISF glucose sensors, comparing materials, center-to-center spacing, edge width, and insertion depth (height).

**Figure 12:**
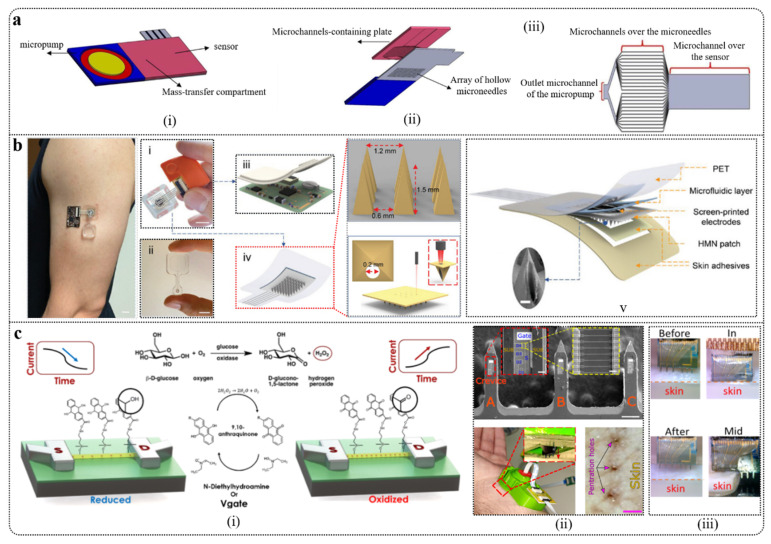
(**a**) Microfluidic system design: (**i**,**ii**) Two different views of our integrated device for regular glucose measurement. (**iii**) Microchannel system for the flow of dialysis fluid [266]. (**b**) MN patch and vacuum system: (**i**) Integration of the MN patch and flexible electronics board. (**ii**) PDMS vacuum generation device. (**iii**) Electronic board of the encapsulation layer. (**iv**) Magnified image of the MN patch. (**v**) Components of the MN patch [260]. (**c**) SiNW-FET and sensor stability: (**i**) Reaction diagram of surface-modified SiNW-FET in the presence of metabolites and oxidases. (**ii**) SEM image of the sensor chip layout (scale bar: 250 μm) and penetration holes on skin after 3D-printed patch detection (scale bar: 650 μm). (**iii**) Penetration in 2 mm pig skin and stability after MN insertion and removal [267].

In early studies, the tip radius and insertion depth of MNs were approximately 15–40 μm and 700–1500 μm, respectively. These dimensions determine the degree of minimally invasiveness and the level of pain. Recent research on MN materials has mainly focused on four types: metal, inorganic, polymer, and hydrogel [203]. Additionally, ISF (interstitial fluid) extraction methods are evolving towards less invasiveness and reduced pain. For continuous glucose monitoring, microneedle sensors and microdialysis sensors are commonly used, often integrating microfluidic systems [266]. One design integrates an implanted microfluidic system using microdialysis technology to periodically and painlessly measure glucose levels in diabetes patients without enzymatic methods. This system includes a piezoelectric micropump, a mass transfer chamber (i.e., hollow microneedle array), and an electrochemical amperometric glucose sensor. During operation, after the microneedle is inserted into the skin, surface tension fills the ISF. The semi-permeable membrane at the microneedle base isolates the ISF from the dialysis fluid. The dialysis fluid flows across the membrane surface, absorbing glucose from the ISF through diffusion, and then is measured by the sensor. Other key components of the device include the mass transfer chamber and piezoelectric micropump (Figure 12a). Each microneedle is 150 μm long, with a microchannel height and width of 60 μm and 50 μm, respectively, and the center-to-center distance between adjacent microneedles is 300 μm. The design features a 24 × 12 microneedle array to reduce patient discomfort. The micropump dimensions are 10 mm × 10 mm × 0.65 mm, and the semi-permeable membrane thickness at the bottom of each microchannel is 15 nm. To control fluid flow, two passive flap valves are incorporated in the piezoelectric micropump. Operating at a frequency of 2 Hz with an input voltage of 7.5 V, the micropump can provide a flow rate of 1 μL/min. The system is compatible with non-enzymatic glucose measurement technologies, making it suitable for painless use by diabetes patients. However, this method requires specialized equipment and technical support, while traditional vacuum-assisted ISF extraction may cause discomfort to the subjects during prolonged waiting periods.

An MN vacuum-integrated platform for continuous sampling and electrochemical sensing of ISF has been proposed (Figure 12b) [260]. The system consists of four main components: a vacuum generation and liquid extraction chamber, a 3D flexible microfluidic module, a laser-drilled microneedle patch, and a PCB circuit board. The platform collects ISF using a finger-driven PDMS system to create negative pressure, with the laser-drilled microchannels on the microneedle patch generating this negative pressure. The vacuum generation system includes the vacuum generation chamber, the liquid extraction chamber, and passive (check) valves. By pressing the chamber, the passive valve at the outlet is activated, and the elastic force from the vacuum generation chamber creates negative pressure, opening the valve between the liquid extraction chamber and the vacuum generation chamber. Once the pressures in the two chambers balance, the passive valve closes, and the negative pressure helps extract ISF into the microfluidic chamber. Compared to existing finger-driven devices, this system offers the advantage of continuously adjusting the internal vacuum pressure. The MN patch consists of a series of conical microneedles, each 1.5 mm long, with an edge width of 0.6 mm and a center-to-center distance of 1.2 mm. The sensor uses a current–time (I-t) method for glucose detection. At low concentrations (20–1000 μM), the sensor shows a sensitivity of 0.254 μA/mM and a detection limit of 5.6 μM; at high concentrations, the sensitivity is 7.1 nA/mM with a detection limit of 0.2 mM. This allows the sensor to effectively monitor glucose levels within the normal blood glucose range (approximately 4.4 to 6.6 mM), consistent with ISF glucose levels. Additionally, the sensor exhibits excellent stability over a 7-day storage period.

The wearable artificial pancreas system based on ISF integrates MN technology, offering a minimally invasive, transdermal solution for continuous metabolite monitoring [267]. This platform utilizes vertical micrometer-scale silicon microneedles and silicon nanowire field-effect transistor (SiNW-FET) arrays for metabolite detection through chemically modified nanowire surfaces (Figure 12c). The system features high sensitivity, selectivity, and low power consumption, operating at a voltage of less than 1 V. It is capable of simultaneously detecting glucose (ranging from 0.35 mM to above 10 mM) and lactate and includes a drug delivery function with an injection rate of 10 nL to 60 μL per minute, suitable for precise delivery of medications such as insulin. The system provides real-time feedback on blood glucose levels and automatically adjusts insulin delivery, mimicking the function of an artificial pancreas. In vitro experiments demonstrate that the sensor reliably monitors glucose and lactate in simulated skin. In vivo trials show that the microneedles penetrate the skin with minimal pain or discomfort, correlating highly with commercial blood glucose meter results, and the sensor signal variance is less than 10%, making it suitable for long-term use. Table 3 summarizes all the works related to wearable glucose sensors based on biological fluids.

Overall, MN-based ISF glucose sensors are trending towards miniaturization, portability, suitability for long-term wear, real-time monitoring, smart features, low cost, and sustainability. However, several challenges need to be addressed: (1) Accuracy and Stability: Ensuring the sensor’s accuracy and stability is crucial. Issues such as sensor–tissue interaction and the dynamic range of glucose concentrations need to be resolved. (2) Biocompatibility: Contact between the sensor and human tissue may cause immune responses or tissue irritation, so the biocompatibility of sensor materials must be considered. (3) Comfort and Wearability: Continuous glucose monitoring requires overcoming challenges related to long-term wear comfort, skin irritation, and pressure. (4) Data Security and Privacy: With the trend towards smart technology, data security and privacy protection are particularly important. (5) Regulation and Clinical Validation: Clinical practice requires stricter regulation and validation to ensure the sensor’s effectiveness and safety.

## 6. Conclusions

Wearable electrochemical glucose sensors based on non-invasive/minimally invasive bodily fluids offer significant advantages, providing a more convenient, comfortable, and accurate monitoring method for diabetes management. However, they still face major challenges: (1) Long-term stability under various environmental conditions: Long-term stability of the sensor in different environmental conditions is a critical challenge. Since wearable sensors need to be worn for extended periods, the materials and components must operate stably over time without being affected by environmental factors. This raises higher demands on the sensor materials. (2) Optimization of mathematical models and algorithms: Mathematical models and algorithms need continuous optimization. Since pH and temperature (T) are related to enzyme activity, the pH and T values need to be strictly controlled under experimental conditions to implement one-order or multi-order calibration algorithms for dynamic glucose calibration in bodily fluids. (3) Microfluidics engineering challenges: Microfluidics faces numerous engineering challenges, including sample pretreatment, system integration, sample volume, real-time detection, and long-term stability. Bodily fluid samples often contain impurities and interfering substances, such as proteins and cell debris, requiring effective pretreatment techniques to improve accuracy and sensitivity. Additionally, microfluidic systems need to integrate multiple functional modules, ensuring coordination between these modules without interference. Handling micro-samples sometimes requires processing larger sample volumes to obtain accurate results, placing high demands on handling and storage units. Real-time detection technologies at the micro-scale face technical difficulties, such as temperature and humidity changes, potentially affecting device performance, leading to long-term stability issues. (4) Miniaturized power supply devices: Miniaturized power supply devices face significant challenges. First, micro-devices need to integrate high-energy-density batteries or supercapacitors within a limited volume, which is often difficult to achieve. Moreover, these power supply devices need to have long charge–discharge cycles and high stability to ensure long-term stable operation. However, current miniaturized batteries still have limitations in terms of lifespan and stability. Power supply devices also need to effectively receive and store energy from external sources and be equipped with efficient charging management systems to ensure safe and stable charging processes.

In recent years, the widespread application of advanced nanomaterials has significantly enhanced the performance of glucose sensors. Researchers have addressed challenges related to energy supply, comfort, portability, and complexity through innovative technologies such as microfluidics, wearable fabrics, and self-powering systems. Non-invasive detection technologies based on biological fluids, combined with wearable devices, allow for rapid sample collection from non-invasive bodily fluids like sweat, tears, saliva, and urine, providing a more convenient management method for diabetes patients and greatly improving their quality of life. Electrochemical glucose sensors based on bodily fluids have tremendous potential, including the following: (1) Non-invasive detection technologies becoming mainstream: The combination of biological fluids and wearable devices will enable rapid and convenient monitoring of glucose levels in the body. (2) Development of advanced nanomaterials: Focus will be on enhancing the stability of nanomaterials, reducing costs, and improving water solubility to create more sensitive and stable sensors. (3) Technology optimization: Improvements in structural design, material performance, and signal processing will further reduce the detection limits of sensors, enhancing stability and reliability. (4) Miniaturization and intelligence: Micro–nano processing and flexible electronics technologies will make sensors more compact and portable, while intelligent connections will enable real-time data monitoring and remote management. (5) Optimization of mathematical models and algorithms: More precise mathematical models and algorithms will improve the accuracy and reliability of glucose measurements. (6) Advancements in microfluidics technology: Innovative microfluidic technologies will enhance sample pretreatment, system integration, real-time detection, and sample handling capabilities. (7) Miniaturization of power supply devices: Integration of higher energy density, long cycle life, and stability into power supply devices will support the long-term use of wearable sensors. (8) Multimodal sensing: Future sensors will be able to simultaneously detect glucose in multiple bodily fluids, overcoming cross-interference and improving sensitivity and specificity. (9) Closed-loop sensing and delivery systems [268,269,270]: This technology will enable automation of sensor placement, sample collection, glucose monitoring, and feedback control, advancing the development of non-invasive/minimally invasive wearable sensors.

## Figures and Tables

**Table 1:** Inherent limitations when applied to biological body fluids.

Principle	Biofluid Interference	Underlying Cause
Amperometry	Reduced current sensitivity Signal drift	Low ionic strength→Higher solution resistance (*R_s_*) [130] Protein adsorption→Electrode fouling [134]
Potentiometry	Nernstian slope deviation Reference electrode instability	Variable Na+/K+ levels→Ionic activity fluctuations [131]
Voltammetry	Peak broadening Redox potential shifts	Fluid viscosity→Altered diffusion coefficients [133]
Impedance	Non-linear Nyquist plots R_ct_-concentration correlation loss	Complex composition→Parasitic capacitances [6]

**Table 2:** Comparison of MN ISF electrochemical glucose sensors in terms of material, center-to-center spacing, edge width, and insertion depth (height).

MNmaterials	Center-to-Center (μm)	Edge Width (μm)	Height (μm)	Ref.
Acupuncture needle	—	80	3000	[257]
Composite ink	—	<4	611 ± 22	[258]
SWCNTs	—	—	640	[259]
PDMS	1200	600	1500	[260]
Polymer	2500	800	900	[261]
PEG-DA	20	160	600	[262]
Au	—	<300	100.16	[263]
PLA	100	200	500	[264]
NgAgo	300 ± 10	—	800 ± 10	[265]
Glass and silicon	300	—	150	[266]

**Table 3:** Performance of wearable glucose sensors based on biological fluids (BFs), material (M), detection (D), extraction volume (EV), sensitivity (S), limit of detection (LOD), linear range (LR), and lifetime (LT).

BF	M	D	E V(μL)	S (μA/mM/cm^2^)	LOD (μM)	LR(mM)	LT	Ref
Tear	GC-COOH	Amperometric method	—	110.92	9.5	0–12	>1 day	[227]
	PBA/HEMA	—	1500	—	—	0.1–0.6	>10 h	[226]
	PET	—	0.4	—	—	—	—	[231]
Saliva	CA/PDMS	—	—	—	—	0.005–1	>5 h	[237]
	Ag/AgCl Oil ink	—	0.5	—	40	100–1400	>60 m	[238]
	Ag/AgCl	Voltammetric method	5	0.1214	5	180–5220	<3000 s	[239]
	Carbon graphite &Ag/AgCl ink	Voltammetric method	10	0.0352	—	48–19,500	—	[240]
Sweat	Graphene/chitosan /PEDOT:PSS	Voltammetric method	0.02–1.0	1.35	—	—	—	[243]
	EFC	—	20	0.02	—	1–5	—	[242]
	GOX/PEDOT:PSS	Amperometric method	2	—	0.075	2–32	—	[244]
	PAA/PAM/PDA	—	4	1576	0.28	0–0.205	—	[245]
	PEDOT:PSS	Cyclic voltammetry	—	325.99 ± 0.8	10.3	0–12	>10 days	[247]
Urine	NiCo_2_O_4_	—	—	3449.14	0.376	1–100,000	—	[250]
	LIG/Ni	—	—	5796.18	0.0152	12–1500	—	[248]
	PLA/PEG	Amperometric method	20	—	197	500–5500	>60 days	[251]
	MCNT/RGO	—	250	—	3.95	0–4000	—	[252]
ISF	MWCNTs/CSF	—	—	288.86	—	0–5000	—	[167]
	—	Differential pulse voltammetry	—	0.549	0.08	25–300	>28 days	[168]

## Data Availability

The raw/processed data required to reproduce these findings cannot be shared at this time as the data also forms part of an ongoing study.
